# Transforming growth factor-β in stem cells and tissue homeostasis

**DOI:** 10.1038/s41413-017-0005-4

**Published:** 2018-01-31

**Authors:** Xin Xu, Liwei Zheng, Quan Yuan, Gehua Zhen, Janet L. Crane, Xuedong Zhou, Xu Cao

**Affiliations:** 1 State Key Laboratory of Oral Diseases & National Clinical Research Center for Oral Diseases & Department of Cariology and Endodontics, West China Hospital of Stomatology, Sichuan University, Chengdu, China; 2 State Key Laboratory of Oral Diseases & National Clinical Research Center for Oral Diseases & Department of Pediatric Dentistry, West China Hospital of Stomatology, Sichuan University, Chengdu, China; 3 State Key Laboratory of Oral Diseases & National Clinical Research Center for Oral Diseases & Department of Oral Implantology, West China Hospital of Stomatology, Sichuan University, Chengdu, China; 4 Department of Orthopedic Surgery, Johns Hopkins University School of Medicine, Baltimore, MD USA; 5 Department of Pediatrics, Johns Hopkins University, Baltimore, MD USA

## Abstract

TGF-β 1–3 are unique multi-functional growth factors that are only expressed in mammals, and mainly secreted and stored as a latent complex in the extracellular matrix (ECM). The biological functions of TGF-β in adults can only be delivered after ligand activation, mostly in response to environmental perturbations. Although involved in multiple biological and pathological processes of the human body, the exact roles of TGF-β in maintaining stem cells and tissue homeostasis have not been well-documented until recent advances, which delineate their functions in a given context. Our recent findings, along with data reported by others, have clearly shown that temporal and spatial activation of TGF-β is involved in the recruitment of stem/progenitor cell participation in tissue regeneration/remodeling process, whereas sustained abnormalities in TGF-β ligand activation, regardless of genetic or environmental origin, will inevitably disrupt the normal physiology and lead to pathobiology of major diseases. Modulation of TGF-β signaling with different approaches has proven effective pre-clinically in the treatment of multiple pathologies such as sclerosis/fibrosis, tumor metastasis, osteoarthritis, and immune disorders. Thus, further elucidation of the mechanisms by which TGF-β is activated in different tissues/organs and how targeted cells respond in a context-dependent way can likely be translated with clinical benefits in the management of a broad range of diseases with the involvement of TGF-β.

## Growth factor: Activation in health and disease

Targeting a critical growth factor involved in bone and other tissue remodeling could help treat osteoarthritis and other skeletal disorders. A team led by Zhou Xuedong from Sichuan University in Chengdu, China, and Xu Cao from the Johns Hopkins University School of Medicine in Baltimore, Maryland, USA, review the ways in which temporal and spatial activation of transforming growth factor-β (TGF-β), a multi-functional signaling molecule, are needed for proper tissue development and regulation of stem cells throughout the body. Looking at the skeletal system in particular, the researchers discuss how TGF-β controls the balance between bone resorption and bone formation. Faulty TGF-β signaling can lead to numerous bone-associated disorders, including rare genetic diseases and metastatic cancers. The authors also summarize clinical efforts to modulate TGF-β with drugs for the treatment of osteoarthritis and other conditions.

## Introduction

The evolution of a multicellular organism into ever more complex life forms needs the establishment of communication and control among individual cells to maintain order in the organism. The basic physiological processes, including proliferation, differentiation, metabolism, and apoptosis, are intricately regulated by a dense signaling network that is elicited by cytokines, growth factors or polypeptide hormones. Among those polypeptide/hormone-induced signals, the transforming growth factor-β (TGF-β) family is particularly important.^1^

TGF-β 1–3 are unique multi-functional growth factors because they are present only in mammals, mainly secreted as a latent complex and immediately stored in the extracellular matrix (ECM).^1, 2^ The biological functions of TGF-β can only be delivered after ligand activation, which is intricately regulated in response to ECM perturbations.^2–4^ Hence, the TGF-β complex functions as a molecular sensor which responds to environmental perturbations by releasing an active TGF-β ligand, to promote or inhibit cell proliferation in a context-dependent manner. More importantly, activation of TGF-β in the right place at the right time is necessary to recruit stem/progenitor cells to participate in the tissue regeneration/remodeling process, whereas sustained abnormalities in TGF-β ligand expression, bioavailability, activation, receptor assemblage/stabilization, or post-transcriptional modifications will inevitably disrupt the normal physiology, and lead to pathobiology of major diseases either through the recruitment of excessive progenitors (as seen in osteoarthritis or Camurati–Engelmann disease), or trans-differentiation of resident cells to unfavorable lineage commitment (as seen in epithelial to mesenchymal transition during cancer metastasis or tissue/organ fibrosis).^1,5–8^

Understanding the mechanisms that underscore the temporal and spatial activation TGF-β, as well as how targeted cells contextually integrate the downstream signaling into coherent responses are essential to elucidate the central role of TGF-β in maintaining stem cell and tissue homeostasis. This may provide new insights into potential treatment of systemic or local disorders that are associated with abnormalities of TGF-β signaling.

## Temporal and spatial activation of TGF-β is essential for tissue homeostasis

TGF-β proteins belong to the TGF-β superfamily, which consists of TGF-β1–3, the activins/inhibins/Müllerian-inhibiting substances (MIS), bone morphogenetic proteins (BMPs), Nodal, growth/differentiation factors (GDFs), and the distantly related glial cell line-derived neurotrophic factors (GDNF) family.^9–11^ TGF-β1–3 are present only in mammals. They are pleiotropic, regulate cell proliferation, migration, and differentiation during embryonic development, and have an essential role in maintaining tissue homeostasis in adults. In mammals, distinct genes encode TGF-β 1–3 isoforms, which are expressed in unique, occasionally overlapping patterns and can perform a variety of distinct functions in vivo.^12–14^ Initially cloned from human term placenta mRNA, TGF-β1 is the most abundant and ubiquitously expressed isoform.^15^ TGF-β1 has been identified in cartilage, endochondral, and intramembranous bone and skin during mouse development, thereby indicating its involvement in the development of these tissues/organs.^16^ TGF-β2, also known as glioblastoma-derived T-cell suppressor factor (G-TsF), was first discovered in human glioblastoma cells. During embryonic development, TGF-β2 is expressed by neurons and astroglial cells.^17^, whereas pathologically it is also involved in tumorigenesis by enhancing cell proliferation and reducing the host immune surveillance against tumor development.^18^ TGF-β3 was first identified from a cDNA library of a human rhabdomyosarcoma cell line. It has an essential role in the development of the palate and lungs, mainly through the regulation of epithelial–mesenchymal interactions during embryonic, fetal, and neonatal development.^12,19^ TGF-β3 is also possibly involved in the wound healing process, orchestrating an orderly migration of dermal and epidermal cells in injured skin.^20^

Although it was discovered more than 30 years ago, TGF-β, as a multi-functional cytokine, is still under major research in various fields ranging from embryonic development to adult organ physiology and pathobiology of major diseases, including cancer, organ fibrosis, cardiovascular diseases, and immunological abnormalities. Unlike most of the growth factors that are ready to function upon secretion, TGF-β is unique in that it is secreted as part of a latent complex that is stored in the extracellular matrix (ECM). Thereby, the magnitude and duration of TGF-β signaling is carefully controlled at many different levels, including the synthesis and activation of latent TGF-β isoforms, receptor activation and stability, and the activation and stability of intracellular Smad molecules and other downstream signaling molecules. Plenty of molecules have been identified as “TGF-β activators” whose mutation will lead to aberrant activation of TGF-β and ultimately pathological phenotypes. Although distal effects from circulating factors have been reported, TGF-β-mediated effects are usually restricted at the sites where the active ligand is released. Therefore, the temporal and spatial activation of this growth factor is critical for its context-dependent physiological effects in vivo. Considering the close relationship of TGF-β and ECM homeostasis, increasing evidence has indicated that TGF-β complex is more like a molecular sensor that responds instantly to ECM perturbations through the release of an active ligand that exerts physiological effects at a cellular level, thus ensuring normal tissue homeostasis.^2^ This section will first elaborate on the molecular basis of TGF-β latency and specific activation pathways that modulate its activation. This section will then further specify how the active TGF-β isoform functions alone or with the cross-talk of other environmental cues, balances the self-renewal of stem cells, assists with differentiation during normal physiological development, and how TGF-β acts as a pro-migratory factor to mobilize adult stem cells from their unique niche to repair damage and maintain normal tissue homeostasis.

### Latent TGF-βs are deposited in ECM upon secretion

TGF-β family members are typically secreted and deposited in the ECM in its latent form, and their biological effects can only be delivered upon ligand activation. TGF-βs contain a characteristic cysteine-knot that is formed from multiple intra-chain disulfide bonds.^9–11^ Take TGF-β1 for example: the precursor peptide contains 390 amino acid (aa), including a signal peptide and a TGF-β1 pro-protein. This pro-protein (361 aa) is processed intracellularly by a furin-like convertase to generate an N-terminal latency-associated peptide (LAP, 249 aa), and a C-terminal mature TGF-β1.^21–26^ Both LAP and mature TGF-β1 form homodimers via disulfide bonds. After secretion, the LAP and TGF-β1 homodimers are further non-covalently associated as the small latent TGF-β1 complex (SLC). LAP-growth factor association is both necessary and sufficient to confer latency of TGF-β1–3, BMP-10, and GDF-8/myostatin. However, for BMP-4, -5, and -7, although LAP and the mature growth factor is also non-covalently associated, the complex is still active.^27^

In most cases, LAP of the SLC is further covalently associated with a latent TGF-β binding protein (LTBP) in the ECM, thus creating the large latent complex (LLC) that functions as an ECM reservoir of TGF-β. The LAP-LTBP association mainly functions to anchor the complex to ECM components such as fibrillin.^2^ LTBP is also involved in the proper folding and secretion of the SLC.^28–33^ To date, four LTBPs (LTBP1–4) have been identified, among which LTBP1, 3, and 4 are able to bind the SLC of all TGF-β isoforms.^34^ Therefore, although TGF-βs are abundant in the ECM.^35–37^, they are secreted and deposited in the latent form, and not able to induce downstream signaling to elicit biological effects.^3,4^

### Latent TGF-βs are activated by various pathways in vivo

Although TGF-β ligand and receptors are ubiquitous in many types of cells, their biological effects are usually restricted at sites where the ligand is activated. Storage of inactive TGF-β in the matrix enables temporal and spatial regulation of TGF-β activation during tissue homeostasis Precise activation of latent TGF-β is a pre-requisite for it to function in the right locations within a specific time frame. In general, the activation of TGF-β requires the release of the LLC from the ECM and further proteolysis/deformation of LAP to release active TGF-β.^38^ Accumulating evidence has shown that TGF-β1 can be activated by plasmin, matrix metalloproteinases (MMPs), thrombospondin-1, lower pH, and reactive oxygen species.^2^ More importantly, TGF-β can also be activated by specific integrins that bind the Arg-Gly-Asp (RGD) sequence of LAPs. The integrin-RGD associate results in a contractile force-dependent conformational change of the latent complex, which releases TGF-β in its active form.^39,40^ In addition, a plethora of soluble extracellular agonists and antagonists coexist at the site where active TGF-β is released and further complicates the temporal and spatial access of the ligands to receptors.^41^

Until now, a variety of TGF-β activators have been reported. Most of these activators are also indicators of ECM perturbations. As TGF-β has profound effects on matrix homeostasis, it has been generally recognized not only as a cellular effector, but also as a potential sensor for environmental perturbations. Here we will describe well-recognized pathways that contribute to the in vivo activation of TGF-β. Inheritable genetic mutations that release excessive TGF-β from the ECM or induce over-production of the ligand will be specifically discussed in section “Genetic mutations in TGF-β signaling components cause bone-associated disorders”.

#### Proteolytic activation

Many proteases including plasmin and matrix metalloproteinases (e.g., MMP-2 and MMP-9) have been identified in vitro as TGF-β activators.^42,43^ Plasmin and MMP-2/9 are the primary enzymes involved in ECM degradation.^44^ Proteases can cleave the covalent bond between LAP and TGF-β peptide in the proLLC, thereby rendering the LLC activation competent. Proteases can also target the protease-sensitive hinge region of LTBP to liberate the LLC, which can then be further processed for activation.^45^ Proteases may directly cleave LAP to release TGF-β in its active form.^46^ The aforementioned enzymatic activation, couples matrix turnover with the generation of active TGF-β to maintain matrix homeostasis.^47,48^ More notably, plasminogen-null animals fail to replicate the pathology of TGF-β1-null animals, and the multisystem pathology of plasminogen-null animals can be alleviated by removal of fibrinogen.^49^ These observations suggest that plasmin is not solely responsible for the majority of the activation of TGF-β1 in vivo.

#### Activation by thrombospondin-1

Thrombospondin-1 (TSP-1) is a complex multi-functional glycoprotein which mediates cell-to-cell and cell-to-matrix interactions during multiple cellular events in a temporally regulated manner.^50–54^ TSP-1 has an important role in the wound healing process, regulating hemostasis, cell adhesion/migration/proliferation, ECM remodeling, and growth factor (e.g., TGF-β) activation.^55^ In addition to tissue repair, TSP-1 is also involved in tissue fibrosis, possibly by activating TGF-β. Either a blockage of TSP-1 activity or deletion of TSP-1 expression can attenuate pathological tissue fibrogenesis.^56–58^

The primary role of TSP-1 in modulating TGF-β activation is observed during injury, under stress, or in other pathologies involved with ECM perturbations. This phenomenon further supports the concept that the latent TGF-β complex embedded in the ECM functions as a sensor to environmental stimuli. TSP-1 will mobilize necessary molecular machineries to release TGF-β in its active form to meet the needs for tissue repair/remodeling, whereas an excessive response to ECM perturbation may super-activate TGF-β and exacerbate adversary effects such as fibrogenesis. Mechanistically, TSP-1 activates TGF-β by binding to specific sequences of the latent complex and inducing a conformational change to release active TGF-β.^59,60^ In the latent TGF-β complex, the RKPK sequence in the receptor-binding region of the mature TGF-β binds to the LSKL sequence at the amino terminus of the LAP, thus enabling ligand latency.^40,61,62^ TSP-1 activates TGF-β through the specific association of its type 1 repeats (TSRs) with LAP and the mature ligand. When the tryptophan-rich motifs (WSxW) present in each of the 3 TSRs of TSP-1 bind to the VLAL sequence in both LAP and the mature TGF-β ligand, it deforms the LAP-TGF-β complex by “inserting” a TSP-1 molecule. In addition, the KRFK sequence in the second TSR of TSP-1 can competitively bind to the LSKL sequence in the LAP and present to the receptor the mature TGF-β domain.^63^ In vivo evidence for the role of TSP-1 in TGF-β activation is shown by the fact that both TSP-1 and TGF-β1 null animals developed strikingly similar pathologies in multiple organs, particularly in the lungs and pancreas. During the perinatal period, administration of the KRFK peptide partially resolved the abnormal TSP-1 depletion phenotypes, specifically airway epithelial hyperplasia and pancreatic islet hyperplasia/acinar hypoplasia. In addition, wild-type mice treated with the LSKL blocking peptide in the perinatal period showed similar features to the TSP-1 knockout phenotype in both the airways and pancreas.^64^ Double knockout of β6 integrin and TSP-1 led to a phenotype different from either single knockout, characterized by cardiac degeneration, severe inflammation, and epithelial hyperplasia, which suggests a potential synergy between β6 integrin and TSP-1 in regulating latent TGF-β activation.^65^

The TSP-1-mediated TGF-β activation is observed in multi-organ fibrosis. Moreover, the expression of TSP-1 is induced by factors such as reactive oxygen species, high glucose, and angiotensin II which are closely associated with systemic diseases that have fibrotic end-organ involvement.^66–69^ Studies using TSP-1 antagonist peptides and diabetic TSP-1 knockout mice have demonstrated that TSP-1 is a major factor inducing fibrotic end-organ complications in diabetes.^58,70,71^ Treatment of diabetic mice with intraperitoneal injections of LSKL improved left ventricular function, and reduced Smad phosphorylation and cardiac fibrosis.^70^ Similarly, treatment with LSKL suppressed urinary TGF-β activity and improved markers of tubulointerstitial injury and podocyte function in diabetic mice.^71^ Moreover, evidence from several studies have demonstrated that TSP-1 can activate alveolar macrophage-dependent TGF-β in bleomycin-induced pulmonary fibrosis animal models, and either CD36 antagonist peptides or TSP-1 can reduce TGF-β activity and ameliorate pulmonary fibrosis.^72,73^

TSP-1-mediated TGF-β activation is also involved in the dermal wound healing process. The phenotype of excisional wound healing in the TSP-1 null mouse is consistent with a decrease in local TGF-β activation^54^, a delay in macrophage recruitment and capillary angiogenesis, and a persistence of inflammation, granulation tissue, and neovascularization.^74^ The TSP-1 null wound phenotype can be largely rescued by topical treatment with the KRFK activating peptide.^74^ KRFK treatment increased the TGF-β levels in these wounds and its effects were blocked by a pan-specific anti-TGF-β antibody. These data suggest that TSP-1 is essential for the local activation of TGF-β during injury and may affect the wound healing process. In addition, subcutaneous implantation of TSP-1-soaked sponges increased levels of active TGF-β and induced fibroblast migration.^75^ Overexpression of TSP-1 in scleroderma and in keloids induces increased TGF-β activity.^76–78^ All these data validate the involvement of TSP-1-induced TGF-β activation in dermal wound healing and sclerosis. However, how to modulate TSP-1 activity to avoid either defective or excessive wound repair processes in vivo remains to be determined.

#### Activation by integrins

**Fig. 1:**
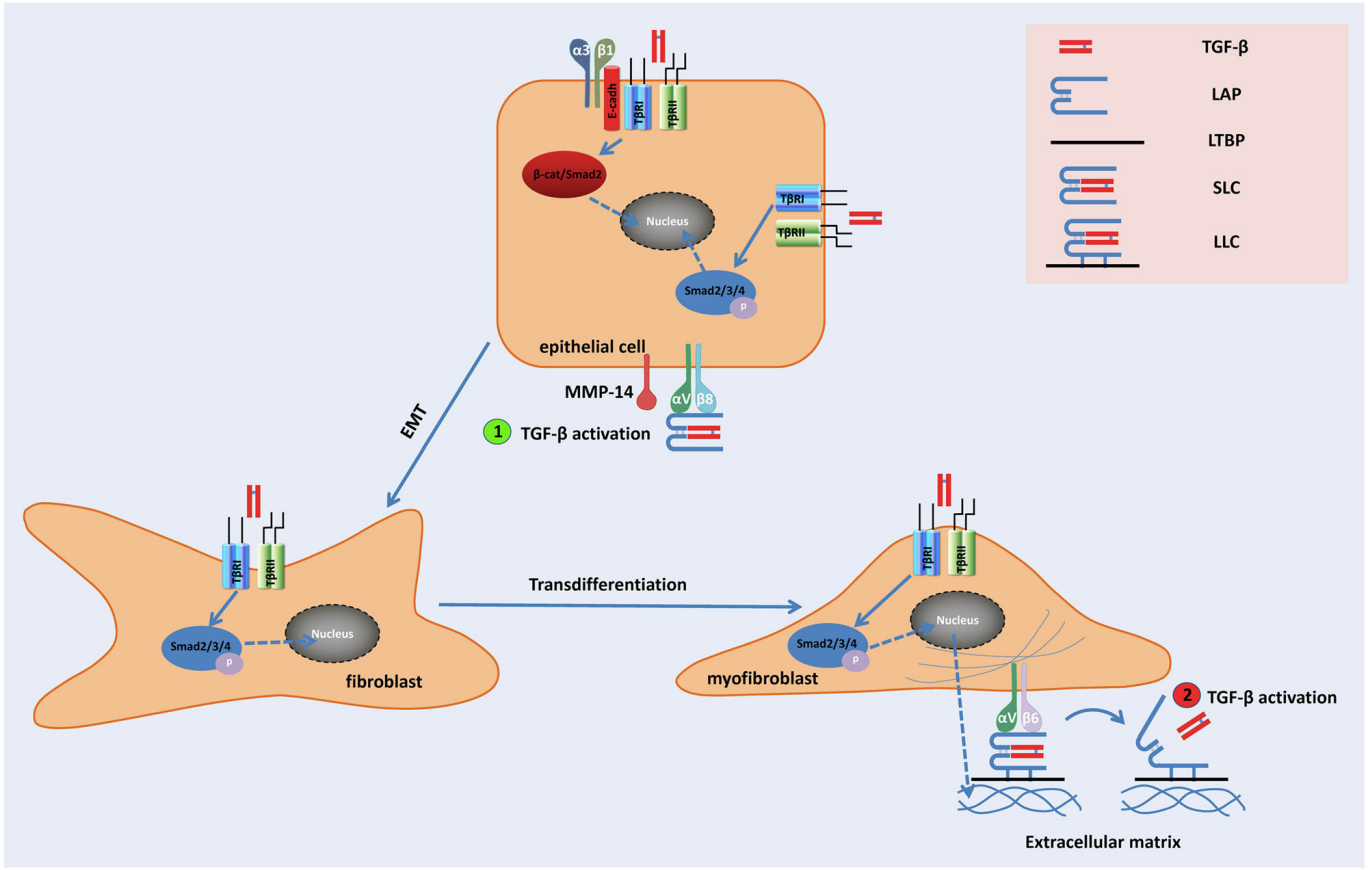
A model of integrin-mediated TGF-β activation during tissue/organ fibrosis. Epithelial cells activate TGF-β by enriching the latent complex through αvβ8-RGD association and recruiting membrane-bound matrix metalloproteinases (e.g., MMP-14) in proximity for further proteolytic cleavage ①. Active TGF-β can act on resident fibroblasts, inducing its trans-differentiation into myofibroblasts, which are the major contributor to excessive ECM (e.g., collagen) deposition and fibrosis. The myofibroblasts can further activate TGF-β in a contractile force-dependent manner through the αvβ6-RGD association ②. The active TGF-β can in turn act on epithelial cells, fibroblasts, and myofibroblasts in a paracrine/autocine manner, and thus form a feed-forward loop for a sustained TGF-β activation and fibrogenesis. Of note, sustained activation of TGF-β can also induce the epithelial–mesenchymal transition (EMT) of epithelial cells with the assistance of integrin α3β1, which forms a complex with TGF-β type I and II receptors (TβRI/II) and E-cadherin, facilitating β-catenin/Smad2 complex formation and nuclear translocation. LAP: latency-associated peptide, LTBP: latent TGF-β binding protein, SLC: small latent complex, LLC: large latent complex.

Integrins are dimeric cell-surface receptors composed of α- and β-subunits.^79^ They have been recently shown to have a central role in TGF-β activation.^80^ Current data show that at least two mechanisms are involved in the activation of latent TGF-β by integrins (Fig. 1). The first proposed mechanism is MMP-dependent. Specifically, integrins are suggested to spatially arrange MMPs, latent TGF-β and the TGF-β receptor in close proximity, which promotes further activation of latent TGF-β by proteolytically cleavage. The second mechanism is proteolytic action-independent, but more closely associated with cell traction forces that are directly transmitted to the LLC via integrin binding. The cellular contractile force can lead to conformational change of the latent complex, thus liberating TGF-β in its active form and/or presenting it to its receptor. It should be noted that both mechanisms are not mutually exclusive, and it is conceivable that cells can use either or both mechanisms at the same time depending on the specific organs or conditions.^81^

α_v_β_6_ was the first integrin that was identified as a TGF-β activator.^39^ The mechanism of activation depends upon a direct interaction between α_v_β_6_ and the RGD amino acid sequence of the prodomains (LAPs). The prodomains of TGF-β1 and TGF-β3 contain an RGD motif that is recognized by α_v_ integrins. Mice with the integrin-binding RGD motif mutation show similar phenotypes to TGF-β1-null mice, such as multi-organ inflammation and defects in vasculogenesis, confirming the essential role of integrins in TGF-β activation.^82^

α_v_β_6_ is normally expressed in epithelial cells at low levels.^83^ Inflammation or injury can increase the expression of αvβ6.^84,85^ Therefore, upregulation of αvβ6 and subsequent TGF-β activation in epithelial cells are believed to be a cellular response to suppress the perturbation such as inflammation. Consistent with the ability of β_6_ integrin to activate latent TGF-β and the pro-fibrotic effects of TGF-β, ^86^ in the mouse model of pulmonary fibrosis induced by belomycin, wild-type mice develop pulmonary inflammation with subsequent fibrosis, whereas integrin β_6_^–/–^ mice show a minor fibrotic response in response to bleomycin.^39^ Moreover, TGF-β-targeted genes in the lungs of integrin β6^–/–^mice are not significantly induced by bleomycin compared to wild-type mice. These data indicate that the inflammatory stimulus upregulate the expression of αvβ6 and consequently induce excessive activation of TGF-β that results in fibrosis. As TGF-β markedly upregulates expression of αvβ6 by primary airway epithelial cells in vitro,^87^ it is likely that bleomycin triggers a feed-forward mechanism for coordinately upregulating integrin expression and TGF-β generation. We suggest that fibrosis is the result of a failure to interrupt this feed-forward loop that is perpetuated by persistent ECM perturbation after injury or inflammation.

Accumulating evidence has suggested the important role of force-resistance ECM in contractile force-dependent TGF-β activation. Activation by α_v_β_6_ integrin requires LTBP-mediated incorporation of TGF-β into the ECM, and the association of the β6 cytoplasmic domain with the actin cytoskeleton.^39,88,89^ Furthermore, contractile force is necessary for TGF-β activation by myofibroblasts.^81^ Thus, tensile force exerted by integrins across the LTBP–prodomain–TGF-β complex is necessary to change the conformation of the prodomain and to free active TGF-β for receptor binding.^81,88^ A recent study by Shi et al. has solved the structure of latent TGF-β and provided mechanistic insights into latency and force-dependent activation by integrins. By using multi- and single-wavelength anomalous diffraction, they found that the two prodomains (LAPs) form a ring-like structure with arms forming a “straitjacket” that fasten each TGF-β monomer. The RGD motifs of LAPs locate to each shoulder, binding to the α_v_ integrins. Upon applied tensile force, TGF-β1 is freed by the opening of the straitjacket, and is subsequently released from the prodomain and activated for receptor binding.^40^ At least four conditions need to be fulfilled to enable the cell traction-dependent integrin-mediated TGF-β1 activation: (1) the presence of the actin cytoskeleton to generate force and/or to provide mechanical resistance, (2) specific integrins that transmit this force on the LLC, (3) incorporation of latent TGF-β1 into the ECM as LLC, and (4) a second anchor point, i.e., an ECM that mechanically resists the cellular traction forces exerted to the LLC. Therefore, the activation of latent TGF-β1 is confined to cells expressing the appropriate integrin in a specific physiological/pathological context. Similar to contractile force-directed activation, a recent study from Coller’s group revealed that intravascular shear force was also able to activate latent TGF-β1 released from platelets.^90^ As TGF-β1 released from platelets during trauma or surgery might also contribute to the transient increase in plasma levels of plasminogen activator inhibitor-1 by activating endothelial cells,^91–97^ the shear force-induced TGF-β1 activation likely coordinates the process of platelet activation to arrest hemorrhage and the transient inhibition of fibrinolysis to allow an unopposed deposition of fibrin at the early stage of hemostasis. The shear force-activation model makes TGF-β1 a potential shear sensor as well as an effector. Hence, TGF-β1 may contribute to the vascular remodeling that occurs in response to changes in shear forces and maintain intravascular arterial shear within a limited range.^98^

In addition to force-directed activation, integrins can also activate latent TGF-β1 with the assistance of protease. α_v_β_8_ is believed to be able to recruit membrane-bound MT1-MMP to the latent complex. This close proximity promotes activation of latent TGF-β1 by further proteolytic cleavage.^99^ Similarly, integrin α_v_β_3_ has been proposed to act as a docking point for MMP-9 in metastatic breast cancer cells,^100^ and for MMP-2 in melanoma cells.^101^ In addition, integrins can cluster with the TGF-β-RII, thereby improving its availability to locally activate TGF-β1. Direct interaction with TGF-β-RII has been demonstrated for α_v_β_3_ integrin upon stimulation with active TGF-β1 using a bioluminescence resonance energy transfer approach^102^ and immunoprecipitation. Interestingly, a similar interaction between different classes of latent TGF-β activator has also previously been suggested:^103^ the cell-surface-associated proteins (CD36 and TSP-1) concentrate latent TGF-β on the membrane where it is subsequently activated by plasmin. This cell surface-enrichment theory might also explain why mice that have null mutations in the genes encoding known protease activators thus far do not demonstrate any phenotype consistent with TGF-β deficiency. It is conceivable that protease activity is intricately modulated by its activators and inhibitors in vivo, as well as the surface concentration of proteases, and a proper spatial arrangement of latent TGF-β1, proteases and TGF-β receptor is a pre-requisite for the in vivo activation of TGF-β1 through proteolytic pathways.

Keeping in mind the two sets of activation mechanisms by integrins may help elucidate some “contradictory” data on integrin-mediated TGF-β activation in different tissues/organs or disease models. The involvement of β integrins in the activation of TGF-β is equivocal, mainly because the deletion of β integrins usually presents contradictory phenotypes in different disease models. Deletion of β6 integrin has been reported to protect mice from bile duct ligation-induced hepatic fibrosis,^104^ whereas global deletion of β3, β5, or β6 integrins or the conditional deletion of β8 integrins in hepatic stellate cells cannot protect mice from carbon tetrachloride-induced hepatic fibrosis.^105^ Because TGF-β activation in a bile duct ligation model is more likely to be contractile force-dependent, β6 anchorage to cytoskeleton is likely essential to ligand activation in the disease model, thereby conveying that β6 deletion is protective. Conversely, in carbon tetrachloride-induced hepatic fibrosis, excessive proteases are released due to extensive cytotoxic damage. In this case, integrin-mediated surface enrichment of proteases may be the major contributor to TGF-β activation, hence single deletion of β subunits is not sufficient to disrupt the superactivation of TGF-β. A similar theory can also explain the observation that integrin β6^−^^/−^ mice have only minor lung fibrosis in response to bleomycin induction.^105^ Because the lungs are a highly contractile organ and its compliance is closely associated with the force-directed activation of TGF-β, β6 deletion directly disrupts intracellular anchorage, and thus may significantly retard the activation of TGF-β and lung fibrosis.

#### Activation by osteoclasts

Latent TGF-β present in conditioned medium can be activated by mild acid treatment (pH = 4.5),^46^ which probably denatures LAP and thus dissociates TGF-β. In vivo, osteoclasts generate a similar pH during bone resorption when an integrin-dependent sealing zone is generated between the bone and the cell.^106^ As the bone matrix deposited by osteoblasts contains abundant TGF-β in its latent form (~200 μg/kg),^37,107^ the acidic environment created by osteoclasts offers an ideal condition for TGF-β activation.^108,109^ Bone-conditioned medium harvested from bone cultures during bone resorption usually contains an increased level of active TGF-β, and the isolated osteoclasts are able to activate bone latent TGF-β in vitro.^8,110^ All this evidence indicates that latent TGF-β from surrounding bone tissue or stored in bone matrix becomes activated and released at this site during the bone resorption process. Alternatively, osteoclasts may also activate latent TGF-β by secretion of proteases in the absence of a low-pH environment. Protease action at a pH higher than the optimum for lysosomal enzyme activity may sufficiently retard enzyme activity to prevent degradation of TGF-β. It is therefore possible that osteoclast-mediated activation of latent TGF-β occurs outside the low-pH resorption lacuna, resulting in the presence of active TGF-β within the immediate environment of the bone resorption site.^109^ As the active TGF-β released during osteoclastic bone resorption is able to induce migration of osteogenic bone marrow mesenchymal stem cells (MSCs) to the bone resorption sites,^8^ osteoclast-mediated activation of TGF-β may therefore represent one of the mechanisms that couple bone resorption to new bone formation. Indeed, our recent study has shown that osteoclast-mediated release of active TGF-β1 is essential for the recruitment of MSCs to the bone resorption site during the parathyroid hormone (PTH)-induced bone remodeling process. By inhibiting osteoclast bone resorption with alendronate, osteoblast recruitment is uncoupled from PTH-induced bone resorption.^111^

#### Activation by reactive oxygen species (ROS)

Another potential mechanism for in vivo activation of TGF-β involves reactive oxygen species (ROS).^112,113^Barcellos-Hoff and her co-workers have shown that ionizing radiation increases the level of active TGF-β in exposed tissues, and that a ROS-generating metal ion-catalyzed ascorbate system is also able to activate recombinant latent TGF-β in vitro.^112,113^ ROS can stimulate the expression and secretion of TGF-β in a positive feedback loop in many types of cells, including hepatic stellate cells and hepatocytes.^114,115^ In addition, low level photodynamic therapy (10 J/cm^2^), which releases free radicals by light activation, has also been shown to increase active TGF-β when applied to cultured smooth muscle cells.^116^ It is currently believed that site-specific oxidation of LAP elicits a conformational change in the latent complex releasing free active TGF-β.^112^ ROS may also indirectly activate TGF-β through MMP activation.^117^ The activation of TGF-β in response to oxidative stress may reflect a need of the human body to produce TGF-β to maintain tissue homeostasis after perturbation such as inflammation. Indeed, LAP/TGF-β1 complex has been proposed to function as an oxidative stress sensor.^118^

### Active TGF-βs bind specific receptors to elicit downstream signaling

Active TGF-β ligands signal by binding and bringing together two transmembrane serine-threonine kinases, known as receptor types I and II.^119^ In vertebrates, seven type I receptors [Activin-receptor like kinases (ALKs) 1–7] and five type II receptors have been identified so far.^120^ TGF-β superfamily ligands bind to and signal through specific type I and type II receptor complexes. Accessory receptors, including the type III receptor, TGF-β RIII (also known as betaglycan) and endoglin, have also been identified.^10,121–124^ Nevertheless, neither betaglycan nor endoglin is directly involved in intracellular TGF-β signaling due to the deficiency of a kinase domain. Instead, they affect the access of TGF-β ligand to its receptors, and consequently modulate the intracellular signaling.^125,126^ Betaglycan binds all three isoforms of TGF-β, with a particularly higher affinity for TGF-β2. However, endoglin binds TGF-β1 and TGF-β3 with identical affinity, and it has weak affinity for TGF-β2.^127,128^

#### Canonical signaling pathways (Smad-mediated signaling) of TGF-β

In most of the context, active TGF-β signals through a canonical (Smad-mediated) pathway. Upon ligand activation, a type II receptor phosphorylates its type I receptor partner, which then transmits the signal by phosphorylation of intracellular downstream substrates, i.e., Smads. Eight Smads (Smad1 to Smad8) have been identified in vertebrates.^129^ They have conserved Mad homology (MH)1 and MH2 domains connected by a linker region. The N-terminal MH1 domain has a β-hairpin loop which can bind to DNA, and the C-terminal MH2 domain mediates interaction with other molecules (e.g., receptors and other Smad isoforms).^130^ The linker region is subject to posttranslational modifications which affect interactions and the stability of Smad molecules. Upon ligand stimulation and subsequent activation by type II receptors, type I receptors transmit intracellular signaling through phosphorylation of downstream effector Smads.^129,131,132^ Specifically, Smad1/5/8 are activated by BMP receptors, whereas Smad2/3 are activated by TGF-β/activin/nodal receptors. These receptor-activated Smads (R-Smads) form heterotrimers with a common Smad (Smad4) shared by the TGF-β/activin/nodal and BMP signaling pathways, and translocate into the nucleus. The R-Smads, except for Smad2 which has two extra sequences inserted in the MH1 domain perturbing its DNA-binding affinity, can bind to preferred DNA sequences. The DNA sequence specificities of R-Smads add further diversity to the transcriptional responses of TGF-β signaling. Complexes of phosphorylated Smad2/3 and Smad4 bind to AGAC or its complement GTCT, known as a Smad-binding element (SBE).^133,134^ However, Smad4-pSmad1/5/8 complexes preferentially bind to GGCGCC or GGAGCC, known as the BMP-response element (BRE).^135–137^ It is noteworthy that although most of TGF-β signaling pathways go through phosphorylated R-Smads, not all transcriptional responses have Smad4 involvement. For example, in cultured epidermal keratinocytes, IκB kinase (IKK) of the classical nuclear factor κB (NF-κB) pathway recruits pSmad2/3 to a specific promoter region that drives cell differentiation.^138^ Data from recent studies also indicate that R-Smads can regulate miRNA processing in a Smad4-independent and RNA-sequence-specific manner by associating with the p68/Drosha/DGCR8 miRNA processing complex.^139,140^

Because the TGF-β superfamily signaling requires the interaction of type I and type II receptors, the interplay between the canonical BMP signaling pathway and the canonical TGF-β/activin signaling pathway has been noted.^141^ The type 1 BMP receptors (ALK2/3/6) + BMPR2 specifically transduce BMP signals; the type 1 Activin receptors (ALK4/7) specifically transduce signals from Activin/Activin-like ligands. In contrast, the type 2 receptors ACVR2A/B are shared between the BMP and Activin pathways and elicit activation of Smad1/5/8 or Smad2/3 in response to BMP or Activin-like ligands, respectively. TGF-β ligands elicit activation of Smad2/3 but do not share any receptors with BMPs or Activin/Activin-like ligands. In addition, TGF-βs and BMPs bind and assemble their receptors in a distinct manner. TGF-β binds TβR-II first and then crosslinks to TβR-I. This pattern was also adopted by activin,^142^ suggesting that TGF-βs/activins assemble their receptors in an ordered manner.^143^ Conversely, the BMPs and GDFs exhibit a much more heterogeneous pattern of crosslinking, with some binding to their receptors in a stepwise manner, whereas others exhibit weak affinity to a single receptor and instead crosslink to TβR-I and -II simultaneously.^144–149^ These findings indicate that the TGF-β superfamily members might differ in how they bind and assemble their receptors into signaling complexes.

Notably, although TGF-β does not share or compete for receptors with BMPs, both strongly induce phosphorylation of Smad1/5/8 in many different cell types, including fibroblasts, endothelial cells, epithelial cells, and epithelium-derived cancer cells.^150–154^ Despite this common phosphorylation event, TGF-β cannot induce BMP-like transcriptional responses. Grönrooset al.^155^ found that although TGF-β was able to stimulate the phosphorylation of Smad1/5/8 in parallel with the classical induction of Smad2/3 phosphorylation, pSmad1/5 and pSmad3 formed complexes readily binding to BMP-responsive elements and mediated TGF-β-induced transcriptional repression on BMP responses. Therefore, Smad3 has an important role in restricting the TGF-β signaling to the canonical transcriptional output and effectively prevents TGF-β from eliciting BMP-like “off-target” responses

The DNA-binding affinity of Smad complexes is not strong. Hence, they need to interact and cooperate with other DNA sequence-specific transcription factors to target the specific downstream genes.^156^ The requirement of DNA-binding co-factors that either activate or repress transcription results in a context-dependent and cell type-specific response.^129,157^ The forkhead-box family member FoxH1 (previously known as Fast1) was the first identified transcription factor that facilitates Smad-mediated transcription. The Foxh1–Smad2/3–Smad4 complex binds to a composite site known as the “activin response element” (ARE) on target differentiation genes in embryonic cells.^158^ Accredited to the advancement from ChIP-seq, various families of DNA-binding transcription factors that interact with Smads have been identified. These transcription factors cooperate with Smad complexes, targeting a specific subset of TGF-β responsive genes for coordinated regulation of cellular activities.^159^ Among the many factors utilizing Smad complexes as transcriptional co-factors, FOXH1,^160^ EOMES,^161^ OCT4,^162^ and NANOG^163,164^ are particularly involved in stem cells, whereas MYOD1 and PU.1 are more relevant to muscle cells and Pro-B cells, respectively. These findings support the notion that the availability of cell type-specific co-factors determine the cellular response to TGF-β signaling by providing context and directing the transcriptional activity of Smad proteins. Smad-mediated assembly of basal transcription machinery is also dependent on chromatin conformation, and thus Smads interact with and recruit various chromatin-modifying enzymes to DNA.^165,166^ Smad2/3 can interact with the histone acetyltransferases CBP/p300 and recruit the basal transcription machinery, thus initiate transcription from the associated promoter.^167,168^ Alternatively, depending on the context, the Smad complex can also recruit histone deacetylases (HDAC1/3/4/5/6) to remove acetyl residues on histone tails, and thus condenses chromatin and represses transcription.^169^ A well-recognized model through which the Smad complex orchestrates with chromatin-modifying enzymes to gain access to DNA by associating with co-transcription factors to maintain stem cell homeostasis will be discussed in section "TGF-β signaling in embryonic stem cells".

In the unstimulated state, Smad proteins interact with components of the Ran GTPase export/import system^170^ and the nuclear pore complex,^171^ resulting in the formation of a highly dynamic equilibrium in which unphosphorylated Smad proteins constantly shuttle between the nucleus and the cytoplasm.^172^ Upon phosphorylation of the R-Smads and the formation of the heteromeric complex with co-Smad in the cytoplasm, the increased import rate and decreased export rate of the trimer lead to its accumulation in the nucleus. This increased nuclear retention is mediated by transcriptional co-factors such as TAZ and YAP, which are the downstream effectors of the Hippo pathway. This cross talk links the TGF-β pathway to the Hippo pathway and sensing of cell density and cell polarity.^173,174^ Protein phosphatases (e.g., PPMA1 or SCPs) can dephosphorylate the R-Smads, leading to the disruption of the trimer, and eventually turn off Smad signaling.^175,176^

Surface receptors are regulated by endocytosis and degraded by SMURF2 and other HECT E3 ligases.^177^ Inhibitory Smads (I-Smad) such as Smad6 and 7 are transcriptional targets of TGF-β superfamily signaling and bind to activated receptors competing with R-Smad binding and recruiting the SMURF ubiquitin ligases, thus establishing a classical negative feedback loop.^178,179^ Activated R-Smad proteins could also be degraded via the proteasome by ubiquitination via HECT E3 ligases such as SMURF1,2, NEDD4L, and WWP2.^180–182^ R-Smad proteins contain multiple PY motifs in the linker region.^181^ Serine/threonine and proline residues of these PY motifs can be phosphorylated by ERK, GSK3,^183^ and CDK8 and 9,^184^ thereby interacting with WW domains of HECT E3. R-Smads are subsequently degraded by the proteasome and the transcriptional activity is terminated. This provides a platform in which the duration of TGF-β family signaling integrates with other pathways such as IGF, FGF, and WNT.

TGF-β signaling can also be fine-tuned by association with other factors. Our recent study has shown that PTH, which regulates calcium homeostasis and bone metabolism by binding to and activating a G protein-coupled receptor, is able to induce the recruitment and co-localization of TβRII with β-arrestin, an adaptor protein involved in PTH receptor endocytosis, thus mediating the internalization of TβRII–PTH1R as a complex in osteoblasts.^185^ The interaction of PTH and TGF-β signaling at the membrane receptor level may have significant physiological importance in maintaining tissue homeostasis, especially in coupling bone resorption to bone formation. We have demonstrated that the anabolic action of PTH on bone is dependent on active TGF-β1 released by PTH-mediated osteoclastic bone resorption.^111^ However, over-production of active TGF-β ligand in the local microenvironment may blunt the migration of MSCs to the bone resorption sites for coupled bone formation.^8^ Through the endocytosis of the TβRII–PTH1R complex, PTH provides surveillance of the over-activation of TGF-β signaling so as to ensure proper MSC migration mediated by local gradient of TGF-β.

#### Smad-independent signaling pathways

The Smad-independent signaling pathways of TGF-β are generally considered as important effector pathways for tyrosine kinase receptors.^131,186,187^ TGF-β activates these non-Smad pathways through interactions of signaling mediators with the type I/II receptors, either directly or through adaptor proteins. The Smad-mediated downstream gene expression may also activate non-Smad pathways. TGF-β can directly activate the Ras–Raf–MEK–ERK/MAPK pathway through the interaction of ShcA and the TGF-β receptor complex. In response to TGF-β, TGF-β type I receptor mediates tyrosine phosphorylation of ShcA, which then recruits Grb2 and Sos, to form a complex, initiating Ras activation and consequently ERK/MAPK signaling cascade.^188^ TGF-β can also activate TAK1 through TRAF6, an ubiquitin ligase, which interacts with the TGF-β receptor complex, leading to induction of p38 and JNK MAPK signaling.^189,190^ TGF-β also modulates the activities of the small GTPase proteins Rho, Rac, and Cdc42, which regulate cytoskeletal organization and gene expression,^191–193^ however the exact mechanism still remains to be explored. TGF-β-activated RhoA can activate its downstream targets ROCK and LIM kinase.^194^ TGF-β activates Akt through PI3K, ^195,196^ and consequently, initiates signaling pathways, e.g., through mTOR, that have roles in cell survival, growth, migration, and invasion.^197,198^ The roles of TGF-β-induced, Smad-independent signaling in stem cells are still unclear and remain to be elucidated.

#### Cross talk with other pathways

TGF-β can cross talk with several other signaling pathways at the level of ligands, receptors, agonists and antagonists, and thus elicits a context-dependent biological effect to meet the specific needs during development or tissue repair.^199^

##### Wnt signaling

Wnt is implicated in stimulation of cell proliferation during embryonal development and tumorigenesis. Key molecules in the Wnt signaling pathway are the transcription factors β-catenin, T-cell factor (TCF), and lymphoid enhancer factor (LEF). Smads form complexes with both LEF1^200^ and β-catenin,^201,202^ which enhance the induction of epithelial–mesenchymal transition (EMT). In addition, Smad7 forms a complex with β-catenin, which is important for TGF-β-induced apoptosis.^203^ The cross talk between TGF-β superfamily and Wnt signaling pathways has an essential role in dictating stem cell homeostasis in concert with combinatorial activities of other signaling pathways. A typical example of how the cross talk between Nodal/Activin/Smad2/3, ERK/MAPK, and Wnt/GSK3β/β-catenin pathways affects the balance of self-renewal and differentiation status of ESCs has recently been described by Singh et al.^204^ Specifically, activation of PI3K/Akt signaling establishes conditions where Activin A/Smad2/3 performs a pro-self-renewal function by activating target genes, such as Nanog. Although in the absence of PI3K signaling, Wnt effectors are activated by ERK targeting GSK3β, and function in conjunction with Smad2/3 to promote differentiation. This signaling paradigm with convergence on Smad2/3 is believed to have far-reaching implications for cell fate decisions during early embryonic development.

##### Parathyroid hormone

Parathyroid hormone regulates calcium homeostasis and bone metabolism by binding to and activating a G protein-coupled receptor. TβRII forms a complex with and phosphorylates the PTH receptor, which modulates the internalization of the receptor complex. Specifically, PTH induces the recruitment of TβRII as an endocytic activator, which phosphorylates the cytoplasmic domain of PTH1R and facilitates PTH-induced endocytosis of the PTH1R-TβRII complex, and consequently results in downregulation of TGF-β signaling.^185^

##### Notch signaling

The Notch pathway specifies cell fate determination during development. TGF-β induces several Notch receptor ligands, including Jagged1,^205,206^ and Notch signaling induces TGF-β.^207^ The cooperation between TGF-β and Notch signaling enhances EMT. However, there are reports that in certain cell types, e.g., esophageal epithelial cells, Notch signaling counteracts EMT by induction of miR200 that targets ZEB and TGF-β.^208^

##### Tyrosine kinase receptors

A major pathway induced by tyrosine kinase receptors is the Ras pathway. Cooperation between Ras and TGF-β signaling is particularly important during EMT.^209^ In hepatocarcinoma cells, TGF-β induces both platelet-derived growth factor (PDGF) and PDGF receptors, which enhances PI3K and β-catenin signaling and promotes the survival and invasion of cancer cells.^210^ Enhanced PI3K signaling also activates Akt, which phosphorylates and activates Twist, promoting EMT.^211^

##### Hippo

The Hippo pathway senses cell density and controls cell growth via the transcriptional regulators TAZ and YAP. TAZ/YAP binds Smad complexes and sequesters them in the cytoplasm in high-density cell cultures, thereby attenuating TGF-β signaling.^173^ Moreover, the Crumbs polarity complex interacts with TAZ/YAP and promotes their phosphorylation and cytoplasmic retention. Disruption of the Crumbs complex enhances TGF-β signaling and promotes EMT.^174^

### Active TGF-βs induce migration of mesenchymal stem cells

A normal tissue repair or remodeling process not only requires the transient amplification and differentiation of adult stem/progenitor cells, but also the proper migration of these stem/progenitor cells to the sites needed.^212–216^ Latent TGF-βs are generally considered as molecular sensors^2^ that respond to perturbations of the ECM by releasing active TGF-βs as pro-migratory factors, thus mobilizing and recruiting adult stem cells to participate in tissue repair/remodeling. Active TGF-βs are released from the perturbed ECM like many other pro-migratory factors in response to injury or inflammation. Although these other factors regulate mobilization of hematopoietic stem cells (HSCs) and epithelial progenitor cells (EPCs),^217–219^ TGF-βs mediate the migration of MSCs from peripheral blood or surrounding tissue to be integrated into the injured/remodeling tissues.

The normal adult bone undergoes continual remodeling by precisely coordinating the activities of osteoblasts and osteoclasts. Osteoblasts derived from bone marrow MSCs deposit calcified bone matrix; while osteoclasts, which are multinucleated cells derived from macrophages/monocytes in the HSC lineage, resorb bone.^220,221^ Factors released from bone matrix during osteoclastic bone resorption orchestrate migration of MSCs to the resorptive surfaces of the bone. Particularly, osteoclastic bone resorption releases and activates TGF-β1 previously stored in the bone matrix, which recruits bone MSCs to the active bone remodeling sites through the canonical pSmad2/3 signaling pathway. TGF-β recruits MSCs in a gradient-dependent manner, i.e., osteoclastic bone resorption induce activation of TGF-β1, which diffuses from the bone resorption site and acts as a chemoattractant for BMSCs. Osteoblastic progenitors sense the TGF-β1 gradient and subsequently migrate to the bone resorption site, where they are induced to differentiate into osteoblasts in response to other environmental factors such as bone matrix-derived insulin-like growth factor 1 (IGF-1).^222^ Interestingly, either over-activation or inhibition of TGF-β signaling that distorts the local TGF-β gradient may impede the migration of MSCs to the normal bone remodeling surfaces. With this hypothesis, we have delineated the pathogenesis of Camurati–Engelmann disease (CED), in which mutations in the LAP cause conformational dissociation,^223^ resulting in increased release of activated TGF-β1 and distortion of the resorption-induced TGF-β1 gradients. Owing to the inadequate recruitment of BMSC to sites of resorption, poor-quality bone is formed with unfilled resorbed areas and haphazard sclerotic areas.^8^ This theory has also been expanded to explain the pathogenesis of osteoarthritis, in which enhanced osteoclastic bone resorption caused by joint instability results in the release of excessive TGF-β1. The pathologically high level of TGF-β1 ligand in the tibial subchondral bone distorts the physiological TGF-β1 gradients, leading to osteoblastic progenitor aggregation in the bone marrow with compromised bone formation capability (“osteoid islet”). The aberrant bone formation in the subchondral bone in turn causes uneven distribution of stress on the articular cartilage, and in a feed-forward manner leads to cartilage degeneration.^6^ In addition, we have also demonstrated in vivo that the anabolic action of PTH on bone is dependent on the active TGF-β1 released during osteoclastic bone resorption. By inhibition of osteoclastic bone resorption with alendronate, the depleted active TGF-β1 released from bone matrix is insufficient to recruit MSCs to the proper resorptive sites, thus impairing the anabolic action of PTH on bone.^111^

Active TGF-β also controls the mobilization and recruitment of MSCs to participate in tissue repair. A recent study of ours has shown that TGF-βs were activated in the vascular matrix in both rat and mouse models of mechanical injury of arteries. The active TGF-β released from the injured vessels induced the migration of MSCs and the cascade expression of monocyte chemotactic protein-1 (MCP-1), which amplified the signal for migration. Specifically, sustained activation of TGF-β was observed in peripheral blood, and Sca1^+^CD29^+^CD11b^−^CD45^−^ MSCs, of which 91% were also Nestin^+^, were mobilized to peripheral blood and migrated to the remodeling arteries. The MSCs were noted to differentiate into endothelial cells for re-endothelialization and myofibroblastic cells to form thick neointima. Intravenous injection of recombinant active TGF-β1 in uninjured mice was also sufficient to rapidly mobilize MSCs into circulation. Blockade of TGF-β signaling with TGF-β type I receptor kinase inhibitor significantly attenuated the mobilization and recruitment of MSCs to the injured arteries.^224^ These findings strongly indicate that TGF-β is an injury-activated messenger essential for the mobilization and recruitment of MSCs to participate in tissue repair/remodeling.

Consistently, another recent study of ours on the pathogenesis of asthma demonstrates the involvement of TGF-β1 in bone marrow MSCs migration. By using a cockroach allergen-induced asthma mouse model, we found increased MSCs and TGF-β1 activation and its downstream signaling in lungs of CRE (cockroach extract)-treated mice. Further in vitro trans-well assay confirmed that TGF-β1 released from allergen-activated epithelium functions as the primary chemoattractant that induces MSCs migration. Consistently, by either intravenous injection of GFP^+^ MSCs (sorted from bone marrow of Nestin-GFP mice) to the CRE-treated mice, or directly immunizing Nestin-GFP mice with CRE, we observed significantly increased accumulation of GFP^+^ MSCs in the asthma airways. Importantly, the airway accumulation of MSCs was significantly attenuated by systemic administration of TGF-β1 neutralization antibody. Taken together, we believe that TGF-β1 is a primary pro-migratory factor released into the circulation from the injured vessels of the CRE-challenged lung tissue. It mediates the mobilization of MSCs to the circulation and further recruits these cells to the perturbed airways in asthma, likely to participate in tissue repair.^225^

Interestingly, one elegantly performed study by Mao’s group has also demonstrated the cell homing capacity of TGF-β3 in the functional regeneration of the articular surface of the rabbit synovial joint.^226^ By replacing the excised proximal humeral condyles of skeletally mature rabbits with cell-free bio-scaffolds spatially infused with TGF-β3-adsorbed hydrogel, weight-bearing and locomotion of rabbits were resumed 3–4 weeks after surgery. Histological and mechanical analysis of the joint revealed that the TGF-β3-infused bio-scaffolds had recruited more cells than did spontaneous cell migration without TGF-β3. As a result, TGF-β3-infused bio-scaffolds were fully covered with avascular hyaline cartilage and integrated with regenerated subchondral bone that had well defined blood vessels 4 months after surgery. On the contrary, TGF-β3-free bio-scaffolds had only scattered cartilage formation with compromised compressive and shear properties. It should be noted the that the lineage of the recruited endogenous cells was not delineated. However, this study further underscores the importance of TGF-β-mediated cell homing in tissue regeneration.

In addition to mobilization and recruitment of MSCs towards wounds, TGF-β also mediates homing of bone marrow-derived human MSCs to glioma stem cells (GSCs).^227^ By using glioma models, Shinojima et al. found that TGF-β attracts BM-hMSCs via TGF-β receptors (TGFβR). Intravascularly administered BM-hMSCs home to GSC xenografts that express TGF-β. BM-hMSCs carrying the oncolytic adenovirus Delta-24-RGD prolonged the survival of TGF-β-secreting GSC xenografts, and this effect was abrogated by inhibition of TGFβR on BM-hMSCs. These data show that TGF-β/TGFβR axis can mediate the tropism of BM-hMSCs for GSCs, and TGF-β may serve as a predictor for patients in whom BM-hMSC delivery could be effective.^227^

### Context-dependent TGF-β signaling balances stem cells self-renewal and differentiation

Stem cells are long-lived cells functioning to make and replenish the differentiated cells that are lost through normal stress and injury. Stem cells are also capable of replenishing themselves, a process known as self-renewal. Extensive efforts have been made to identify factors that determine the self-renewal and differentiation of stem cells. Stem cells receive signals from their surrounding environment (niche) and galvanize intracellular transduction pathways, which deliver information to its genome via activated transcription factors. These transcription factors cooperate with co-activators and chromatin remodelers, intricately balancing the proliferation and cell fate of stem cells. In addition to pro-migratory effect, the pleiotropic effects of TGF-β have an essential role in balancing the self-renewal and differentiation of stem cells. Because TGF-β is abundantly stored in ECM in the latent form, its temporal and spatial activation and intracellular communication with other signaling pathways (e.g., Wnts signaling) should always be considered, so as to better delineate its context-dependent role in the determination of cell fate.

#### TGF-β signaling in embryonic stem cells

Embryonic stem cells (ESCs) are pluripotent stem cells derived from the inner cell mass of the blastocyst.^228^ ESCs can differentiate into all cell types in the body (pluripotent), whereas adult stem cells can generate only a limited number of cell types (multipotent). In addition, ESCs are capable of proliferating indefinitely.^229^ Hence, ESCs are useful tools for both research and regenerative medicine. Many of the responses of stem cells to TGF-β family ligands are regulated by Smad-mediated transcription activation or repression of key genes. Smads cooperate with master regulators of cell differentiation or pluripotency.^160,162–164,230–235^ In stem cells, some genes are in an active state within the euchromatin, and their Smad binding sites are accessible to incoming Smad4–RSmad complexes. In this case, TGF-β- or BMP-activated Smads increase or decrease RNA polymerase II (Pol II) action and the transcriptional magnitude of these genes. Nodal signaling modulates cell homeostasis (e.g., SerpinE1) or Smad pathway feedback related genes (e.g., Smad7) in this mode of action. Nodal signal-driven Smad complexes, with the assistance of other DNA-binding cofactors, readily bind to the Smad binding sites, thus upregulating or downregulating the basal activity of these genes. However, most genes that control master regulators of stem cell differentiation are in a quiescent but “poised” state, which can be switched to rapid transcription in response to differentiation signals given the chromatin repressive marks are erased. The nature of the inaccessible poised state implies that activation of ESC differentiation genes by the TGF-β/Smad pathway may be different from that of those readily accessible homeostasis genes. In general, chromatin structure modification that allows the access of pSmad2/3–Smad4–FOXH1 complex to the AREs is pre-requisite for TGF-β/Smad regulation. A typical model explaining how Smad complexes gain access to the AREs of poised master regulator genes has been proposed recently.^236^ Goosecoid (*Gsc*) and *Mixl1* are two master genes for mesendodermal differentiation of ESCs. The promoters of these two genes are “poised”, with Pol II being paused at the transcription start site and kept from active transcription by a chromatin compacting complex of H3K9me3 and HP1. In response to Nodal/Activin signaling, the downstream pSmad2/3 forms a complex with tripartite motif 33 (TRIM33, also known as TIF1g/ectodermin), and elicits an active chromatin conformation with an added acetylation mark at histone lysine 18 by histone acetyltransferase p300. The pSmad2/3–TRIM33 complexes then translocate to the nucleus, recognize histone marks, displace HP1, and consequently allow the access of Smad4–pSmad2/3 to the AREs within the *Gsc* and *Mixl1* promoters.^236^ The complex further recruits additional transcriptional regulators, such as FOXH1, further generating the requisite active chromatin conformation and initiating *Gsc* and *Mixl1* transcription. In parallel, Activin/Nodal regulate genes involved in essential cellular functions and homeostasis (such as Smad7 and SerpinE1, which are not in a “poised” state) in a TRIM33-independently way, in which pSmad2/3–Smad4–FOXH1 directly binds the AREs of these genes.^129^ The result of these events is that Nodal switches *Gsc* and *Mixl1* from the poised state to the activated state, and by orchestrating with other induced functional genes, triggers mesendodermal differentiation. In addition, TRIM33 has been observed to mediate Smad4 ubiquitination,^237,238^ thus probably providing a negative feedback activity for the inactivation of Smad4 and signal turnover. Specifically, the Pol II kinases CDK8 and CDK9 phosphorylate Smads complexes at an interdomain linker region to activate transcription. In the process, ubiquitin ligases recognize the phosphorylated linker, leading to proteasome-mediated turnover of Smad proteins and signaling attenuation.^184,239^ In addition, Smad4 can be directly inactivated by poly-(ADP)-ribosylation, which provides another mechanism for decommissioning Smad4 in transcriptional complexes.^240^

The regulation model of Smads-dependent TGF-β singling may help to better understand the contextual role of TGF-β in balancing stem cell pluripotency and differentiation. The “core transcriptional factors” NANOG, SOX2, and OCT4 form an interactive network that induces pluripotency in ESCs.^241,242^ This triad mediates chromatin-modifying complexes to establish repressive marks coexisted with activating marks that poise chromatin for abrupt transcription of differentiation genes.^242^ BMP signaling directs Smad1 to co-occupy the genome with leukemia inhibitory factor (LIF)-activated signal transducer and activator of transcription 3 (STAT3), OCT4, SOX2, and NANOG at sites with activating mark H3K4me3, and thus stimulates self-renewal of ESC.^229^ The consequently activated genes, including *Oct4*, *Sox2*, *Nanog*, and *Id3*,^243^ in turn form a feed-forward cycle. In response to Nodal, the OCT4 complex also activates Nanog and the Nodal negative feedback regulators, Smad7 and Lefty1, Lefty2,^162^ by directing Smad3 to neighboring sites,^162^ and thus maintains the self-renewal and pluripotency of ESCs. In the absence of the pluripotency enforcing factor LIF, ESCs respond to autocrine signals and differentiate into mesendodermal cells of the primitive streak and ectodermal cells. Nodal signaling drives mesendodermal differentiation by inducing the expression of poised *Gsc* and *Mixl1* through the pSmad2/3–TRIM33-mediated mechanisms as detailed above.^236^ The resulting induction of *Gsc* and *Mixl1* commits primitive embryo cells to mesendodermal fates.^236^ In summary, BMP activates Smad1, which co-occupies the genome with LIF-activated STAT3 and the core pluripotency triad OCT4–SOX2–NANOG, thus stimulating ESCs self-renewal. When contextual self-renewal signals attenuate, the poised chromatin marks provide an entry point for Smad3 complexes to activate differentiation genes.

Undoubtedly, signaling cross talk between Smad2/3 pathway and other signaling pathways also have an important role in dictating the stem cell homeostasis. The complex cross talk between Nodal/Activin/pSmad2/3, ERK/MAPK, and canonical Wnt/GSK3β/β-catenin pathways^204^ has provide a paradigm for cell fate decisions during early embryonic development. Therefore, even though stem cells have receptors which enable them to respond to TGF-βs and other growth factors, their responses are determined by the integration of both intrinsic (e.g., distinct master regulators present during development) and extrinsic (e.g., ligand activation, competition or cross talk of various niche factors) factors. As TGF-β and BMP antagonize each other and cross talk with other pathways, even subtle differences can elicit profound or contradictory effects. It is now well-recognized that the master regulators expressed by a specific stem cell lineage decide the genes to be regulated by pSmad2/3–Smad4. The concept may be especially pertinent in explaining why stem cells at distinct developmental stages may respond differently to a collection of extracellular cues. Because the abundance of cell-type-specific master genes increases upon differentiation, the master regulators at a specific lineage stage will competitively interact with Smad2/3,^242^ and ultimately lead to a context-dependent phenotype. In addition, some master regulators may themselves be targets of a signaling pathway, the order in which a cell receives a series of signals also matters. Hence, a spatial and temporal profiling of signaling at the single-cell level is necessary and will lead to promising findings.^244^

#### TGF-β signaling in tissue-specific stem cells

Adult stem cells are undifferentiated cells found throughout the body after development. Some adult stem cells are perpetually active, such as intestinal stem cells, whereas others are quiescent, such as stem cells of the hematopoietic system, hair follicles, and mammary gland. Upon specific environmental stimulations, quiescent stem cells can re-enter the cell cycle in response to specific environmental cues, and give rise to lineage-specific progenitors which then differentiate to make functional tissue. TGF-β superfamily members participate in most of these steps, and universally in most tissues. TGF-β superfamily members balance active proliferation and reversible cell cycle exit, thus maintaining reservoirs of stem cells that respond quickly to external changes. Comprehensive coverage of TGF-β superfamily signaling in all adult stem cells is beyond the scope of this review. Here, we discuss briefly only a few of the well-studied adult stem cells whose proliferation and quiescence have been shown with clear TGF-β involvement. Of note, since we propose that the bone remodeling process is a representative model to demonstrate how TGF-β signaling orchestrates with other environment-derived cues to determine the fate of MSCs at the correct sites, the role of TGF-β as a coupling factor for site-directed differentiation of MSCs/progenitors during the bone remodeling process will be particularly discussed in section "TGF-β is the major coupler of bone resorption to formation".

##### Mesenchymal stem cells

Mesenchymal stem cells are multipotent cells existing in various adult tissues including muscle, adipose tissue, connective tissue, bone marrow and teeth, blood, placenta and umbilical cord.^245,246^ The differentiation potential of MSCs depends on the niche where they locate. Proliferation of human MSCs can be stimulated by Wnt or TGF-β signaling.^247–249^ TGF-β1 induces Smad3-dependent nuclear accumulation of β-catenin, thereby stimulating MSC proliferation. On the other hand, BMP2 antagonizes Wnt3a signaling and inhibits proliferation of MSCs through interaction of Smad1/5 with Dishevelled-1.^250^ In addition to proliferation, TGF-β signaling also directs the differentiation fate of MSCs.^251^ BMPs can induce differentiation of MSCs into chondroblasts or osteoblasts in vitro. TGF-β and activin also promote chondroblast differentiation at early stages, whereas TGF-β inhibits osteoblast maturation at late stages in differentiation.^251^ Hence, inhibition of TGF-β/activin signaling strongly enhances osteoblast maturation.^252^ These inhibitory effects of TGF-β/activin signaling on MSC differentiation are possibly mediated by induction of expression of inhibitory Smads, such as Smad6, which in turn represses BMP signaling.^253^ In addition, BMP7 has been shown to induce the generation of brown fat from MSCs in the absence of the normally required hormonal induction.^254^ TGF-β is also involved in cardiomyocyte lineage differentiation of MSCs. In human MSCs, TGF-β treatment induces the expression of cardiomyocyte markers including α-smooth muscle actin, myocardin, and calponin 1, along with the Notch ligand, Jagged 1. Increased expression of these genes is Smad3- and Rho kinase-dependent. Prevention of Jagged 1 expression blocks the expression of cardiomyocyte genes, suggesting that Jagged 1 has an important role in TGF-β-induced expression of cardiomyocyte marker genes.^255^ These studies implicate that the microenvironment is critical for the induction of MSC differentiation into different lineages. Considering the complexity of the bone marrow niche and the involvement of multiple cell-secreted or bone matrix-derived factors in the determination of cell fate, it is more likely that TGF-β functions as a pleiotropic growth factor that reactivates quiescent stem cells into a transient amplification in response to environmental cues (e.g., ECM perturbation). In the meantime, TGF-β acts as a pro-migratory factor recruiting MSCs to the sites where the cell fate is ultimately determined in consultation with other niche-specific factors to meet the tissue need.

##### Hematopoietic stem cells

HSCs are stem cells found in bone marrow, being able to differentiate into all blood cell types. TGF-β signaling has an important role in regulating the quiescence of HSCs.^256^ In cell culture, TGF-β signaling-deficient HSCs have a higher proliferative capacity, whereas the quiescence and maintenance of HSCs depend on TGF-β signaling.^257,258^ Furthermore, the response of HSCs to TGF-β stimulation is biphasic. High concentrations of TGF-β inhibit HSC proliferation, whereas low concentrations of TGF-β stimulate its proliferation.^259^ As TGF-β is produced in a latent form by various cells, the mechanism of activation is critical for the regulation of HSC quiescence. Non-myelinating Schwann cells have been shown to mediate activation of TGF-β, suggesting that glial cells maintain HSC quiescence by limiting activation of latent TGF-β as components of a bone marrow niche.^260^ Notably, each subtype of HSCs responds distinctively to the TGF-β signaling. For example, TGF-β signaling leads to different effects on myeloid-biased (My-) and lymphoid-biased (Ly-) HSC subtypes.^261^

##### Neural stem cells

Neural stem cells (NSCs) are stem cells giving rise to neural progenitor cells and eventually to neurons, astrocytes and oligodendrocytes.^262^ Targeted inactivation of TGF-β type II receptor gene in the mid/hind brain in the developing stage enhanced the self-renewal of mouse NSCs, resulting in an enlarged midbrain. In the meantime, inactivation of TGF-β type II receptor was accompanied with ectopic expression of FGF and Wnt ligands suggesting that TGF-β signaling may control the size of the midbrain by antagonizing FGF and Wnt signaling, and consequently inhibits NSC self-renewal.^263^

In the adult central neural system, NSCs reside in the sub-granular zone of the dentate gyrus and in the sub-ventricular zone adjacent to the lateral ventricles.^264^ Neurogenesis in this region is regulated by different factors at the level of cell proliferation, fate determination, and survival. TGF-β signaling has an important role in the maintenance and proliferation of NSCs.^263,264^ In the case of brain lesions or neurodegeneration, TGF-β1 is upregulated and activated in astroglial, neuronal and microglia cells,^17,265–267^ and it coordinates cellular responses associated with either beneficial or detrimental effects on the neurogenesis depending on the cellular context.^268–271^ For example, TGF-β is able to induce the synthesis of type 1 Plasminogen activator inhibitor by astrocytes through Smad3-dependent pathway, and thus protects hippocampal, cerebellar, and cortical neurons against *N*-methyl-d-aspartate toxicity.^272^

##### Hair follicle stem cells

Epithelial hair follicle stem cells (HFSCs) are stem cells residing in a specific niche of the hair follicle, referred to as the bulge.^273^ In adult mice, hair follicles undergo dynamic, synchronized phases of growth (anagen), degeneration (catagen), and rest (telogen). Throughout the telogen phase, HFSCs are quiescent. Their quiescence is maintained in part by BMPs provided from the inner layer of non-stem niche cells^274^ and from surrounding dermal fibroblasts and adipocytes.^275^ In the normal niche, BMP signaling must be transiently lowered in favor of transient amplification and lineage commitment of HFSCs, but then it must be restored to the normal level to maintain the quiescence of HFSCs. In addition to BMP, TGF-β also has a role in the telogen phase through induction of apoptosis.^276,277^ Of note, targeted inactivation of Tgfb1, 2, and 3, respectively, results in differential effects on embryonic hair follicle development. Tgfb2^−/−^ mice exhibit a profound delay in hair follicle morphogenesis, characterized by a 50% reduced number of hair follicles.^278^ Mechanistically, TGF-β2, which is primarily produced by dermal papillae, possibly stimulates HFSC proliferation by counteracting BMP-mediated quiescence in the niche.^244^ Moreover, TβRII-deficient HFSCs display elevated pSmad1 and BMP signaling and delayed hair cycle entry, further suggesting the antagonism of TGF-β and BMP signaling in the determination the HFSC proliferation and cell fate. The antagonism between TGF-β and BMP signaling is probably through Tmeff1, which can block BMP2-mediated mesoderm induction in *Xenopus* embryos.^279^ Induction of TGF-β2 signaling and inhibition of BMP signaling in activated hair germ progenitors are normally accompanied with Tmeff1 upregulation,^244^ and TβRII mutation diminishes Tmeff1. Knockdown of Tmeff1 in wild-type HFSCs leads to abrogation of TGF-β2-mediated suppression of BMP signaling, and delays hair follicle regeneration. Moreover, Tmeff1 diminishes the response of wild-type HFSCs to BMP signaling in vitro, in a fashion similar to that of TGF-β2.^244^

##### Skeletal muscle stem cells

Muscle stem cells (MuSCs) are stem cells isolated from skeletal muscle with myogenic potential in response to environmental cues.^280–282^ In the adult, quiescent MuSCs reside between muscle fibers and surrounding basement membranes. BMP4 signals from emerging tendons impact the behavior of a subpopulation of dividing MuSCs at the tips of fetal skeletal muscle.^283^ Upregulation of BMP signaling promotes proliferation of MuSCs, whereas blockade of BMP signaling results in fewer MuSCs.^283^ Upon muscle injury, quiescent MuSCs proliferate and differentiate into myoblasts, and fuse to form de novo multinucleated myofibers.^284^ As the muscle ages, its regenerative capacity declines, possibly due to diminished activation of the Notch pathway.^285,286^ In addition, aged muscle produces excessive TGF-β, which induces erroneously high levels of pSmad3 in resident MuSCs and disrupts their regenerative capacity, whereas attenuation of canonical TGF-β signaling in aged, injured muscle restores MuSC activity.^287^ These findings indicate a shift from active Notch to active TGF-β/pSmad3 signaling in the MuSC niche with age, and the antagonism between TGF-β and Notch has an essential role in controlling MuSC proliferation. Of note, active Notch reduces TGF-β/pSmad3-dependent upregulation of cyclin-dependent kinase (CDK) inhibitors p15, p16, p21, and p27.^287^ These findings suggest that an age-specific interaction between TGF-β and Notch signaling controls CDK inhibitor levels in MuSCs and in turn governs tissue regenerative capacity upon muscle injury.

Taken together, the knowledge regarding how TGF-β signals acts with other contextual environmental cues to determine stem cell fates is not only fundamental to stem cell biology, but could useful for regenerative medicine. As the goal of regenerative medicine is to replace malfunctioning cells/tissues/organs with the competent ones, targeted manipulation of these signals to direct stem cell differentiation into specific cell type will undoubtedly contribute to future applications of stem cells in regenerative medicine.

### Inhibition of TGF-β enhances reprogramming of somatic cells

Adult somatic cells can be forced to reprogram into induced pluripotent stem cells (iPSCs) by ectopically expressing certain transcription factors. The classical iPSC techniques was pioneered by forced expression of the four “Yamanaka factors”, *Oct4*, *Sox2*, *Klf4*, and *c-Myc* in the mouse embryonic fibroblasts and thus reset the differentiation clock of these cells back to the pluripotent state equivalent to a blastocyst.^288^ Many other reprogramming techniques or conditions have been developed afterward, mainly aimed to decrease the risk of genomic insertions of exogenous reprogramming factors or to increase the efficiency of reprogramming process. Because the four “Yamanaka” factors orchestrate to inhibit the TGF-β signaling pathway that induces epithelial–mesenchymal transition (EMT) through Snails, factors that antagonize TGF-β signaling are believed to enhance reprogramming. Indeed, small molecules that can selectively inhibit TGF-β type I receptor kinases enhances iPSC induction and can replace the requirement of Sox2 for iPSC induction. Inhibition of TGF-β signaling in partially reprogrammed iPSCs even induces Nanog expression and ultimately promotes full reprogramming.^289–291^ Like small molecules, Smad7, one of the I-Smad proteins can also replace *Sox2* to enhance the reprogramming process.^292^ Conversely, either treating reprogramming iPSCs with TGF-β, or introducing an activated TGF-β type I receptor decreases reprogramming efficiency.^290,293^ Furthermore, expression of miRNAs that inhibits TGF-β- signaling and EMT enhances iPSC reprogramming.^293–295^ Therefore, TGF-β signaling suppresses somatic cell reprogramming possibly by induction of EMT, although it is important for ESC self-renewal. On the contrary, BMP signaling can induce the mesenchymal–epithelial transition (MET) process, a reversal process to EMT, and thus counteracts TGF-β stimulation in some contexts and promotes reprogramming into iPSCs.^292,296^

## TGF-β is the major coupler of bone resorption to formation

The musculoskeletal system is dynamic in that it undergoes continual adaptations during vertebrate life to attain and preserve skeletal size, shape, microstructure and to regulate mineral homeostasis. In addition, its matrix is a major reservoir of growth factors whose bioavailability and temporal and spatial activation modulate the balance between normal physiology and pathology of tissue/organs involved. Skeletal homeostasis is maintained by intricate regulation of the bone remodeling process. A typical bone remodeling cycle consists of three distinct phases: (a) initiation phase, during which osteoclasts are formed and resorb damaged bone; (b) reversal phase, the transition of osteoclast to osteoblast activity; and (c) formation phase, when osteoblasts rebuild an equivalent amount of bone to that resorbed.^1^ Termination of osteoclast bone resorption and recruitment/differentiation of MSCs are generally recognized as essential steps in the reversal phase.[1] Our recent findings, in combination with those from others, have clearly demonstrated that the bone remodeling process is an optimal model to unveil the mechanisms of how pro-migratory factors present or released in the stem cell niches are orchestrated to mobilize/recruit stem cells/progenitors for a rapid but also transient amplification and differentiation, thus maintaining or restoring homeostasis of involved tissues/organs.

### Bone remodeling is the driving force for the evolution of the terrestrial skeleton

Multiple adaptations were needed for animals to evolve from aquatic to terrestrial life, and a coupled bone remodeling is critical for this process. Osteoblastic bone formation exists in all vertebrates. However, osteoclasts and bone resorption only occur permanently in amphibians and terrestrial animals; whereas the expression of primitive bone resorbing cells and/or osteoclasts are species-dependent in fish, occurring at either specific developmental stages or in times of physiologic stress, such as transitioning from sea to either brackish or fresh water.^297–300^ A major skeletal difference between aquatic and terrestrial animals is that terrestrial vertebrates have to carry their body weight. A lighter skeleton affords a quick, efficient ability to enhance survival. Bone remodeling, with the evolutionary development of osteoclasts, therefore solved the issues of skeletal weight and a mechanism to regulate mineral metabolism through bone resorption.^297^ However, to continue to maintain the integrity of the skeleton, temporal and spatial coordination of osteoblast bone formation with osteoclast bone resorption also became essential in terrestrial vertebrates. Quite a few cells have been identified to participate in the highly coordinated bone remodeling process.

The osteocyte is the most abundant cell population in mineralized bone tissue.^301^ It has been considered as the prominent candidate to sense dynamic changes in mechanical loading, and to initiate the remodeling process. Osteocytes are cocooned in fluid-filled cavities (lacunae) within the mineralized bone,^301^ and possess long dendrite-like processes that extend throughout canaliculi (tunnels) within the mineralized matrix. Osteocytes interact with each other and osteoblasts via these dendrite-like processes,^302^ functioning as a mechanosensor to coordinate activities of other bone cells.^303^ Recent studies have revealed that osteocytes expressed higher concentrations of receptor activator of nuclear factor kappa B ligand (RANKL) than mature osteoblasts and bone marrow stromal cells, further underscoring the essential role of osteocytes to initiate bone remodeling via osteoclastogenesis.^304,305^ In addition, osteocytes regulate bone remodeling by expressing sclerostin (SOST),^306–309^ which negatively regulates bone formation by inhibiting BMP and WNT signaling in osteoblast lineage cells.^310–312^

Other cells present in the bone marrow also dynamically cross talk with osteoblast and osteoclast via either direct cell contact or soluble signals, thus modulating bone homeostasis during normal physiology.^313^ Mature B cells produce 50% of total bone marrow-derived osteoprotegerin (OPG), a decoy receptor for RANKL. Indeed, mice lacking B cells present an osteoporotic bone phenotype.^314^ T cells have been reported to promote OPG production by B cells, possibly through CD40 ligand (CD40L) to CD40 co-stimulation.^314^ Consequently, T-cell-deficient nude mice, CD40 KO mice, and CD40L KO mice display diminished bone marrow OPG production and osteoporotic phenotype.^314^ Megakaryocytes derived from HSCs can also express RANKL and OPG and secrete an unknown soluble anti-osteoclastic substance,^315^ and thus participate in the regulation of bone remodeling. Osteal macrophages (Osteomacs) are a special subtype of macrophage residing on or within endosteal and periosteal surfaces. Osteomacs have an important role in musculoskeletal development, homeostasis and repair.^316^ Osteomacs form a canopy which generates a unique microenvironment to facilitate “coupled” osteoclast resorption and osteoblast formation in a temporary anatomical structure known as “basic multicellular units” (BMUs).^317^ Osteomacs are required for full functional differentiation of osteoblasts in vitro. Depletion of macrophages results in complete loss of osteoblast bone-forming surface evidencing that osteomacs are also required to maintain mature osteoblasts.^318^

### Mesenchymal stem cells are recruited by bone matrix TGF-β to couple bone resorption and formation

Osteoblasts are derived from bone marrow MSCs (also referred to as bone marrow stromal cells or skeletal stem cells).^221^ Ex vivo mouse MSCs can be identified based on the absence of hematopoietic and endothelial markers and the presence of PDGFRa.^319–321^ PDGFRa^+^ Sca-1^−^CD45^−^Ter119^−^cells also express high levels of HSC niche factors *Cxcl12* and Stem cell factor (*Scf*),^319,321^ hence they not only contribute to osteoblast and adipocyte lineage,^321^ but also constitute the cellular component of the HSC niche. Cells that express the Nestin-GFP transgene (Nes-GFP) contain all of the mesenchymal progenitor activity (fibroblastic colony forming units; CFU-F) in mouse bone marrow.^322,323^ These Nes-GFP^+^ cells segregate with distinct vessels in vivo. The Nes-GFP bright cells, which exclusively locate along arterioles, are much rarer than the reticular Nes-GFP dim cells largely associated with sinusoids. The Nes-GFP bright cells are quiescent, contain the most CFU-F, and closely associate with HSC quiescence and maintenance in the bone marrow.^322^ Notably, a recent study reported that the number of both Nes-GFP bright and dim cells significantly decreased in adult endochondral bone, and only a small number of Nes-GFP dim cells were detectable in the adult bone marrow.^324^ These data indicate that Nestin^+^ MSCs may be more relevant to the endochondral ossification during development rather than the skeletal remodeling process in adult, or the expression of Nestin is transient in the MSCs. Another recent study by the Morrison group has demonstrated that Leptin Receptor (LepR) is a marker that highly enriches bone marrow MSCs accounting for 94% of adult bone marrow CFU-Fs.^325^ LepR^+^ cells are Scf-GFP^+^, Cxcl12^−^DsRed^high^, and Nes-GFP dim. They emerge postnatally and give rise to most bone and adipocytes formed in adult bone marrow. LepR^+^ cells are normally quiescent, but they transiently proliferate to participate in bone regeneration after irradiation or fracture.^325^ Therefore, LepR^+^ cells may represent the major cellular source of bone and adipocytes in adult bone marrow, and possibly account for the largest proportion of MSCs that participate in the bone remodeling process and maintain the HSCs in adult bone marrow.^326^

**Fig. 2:**
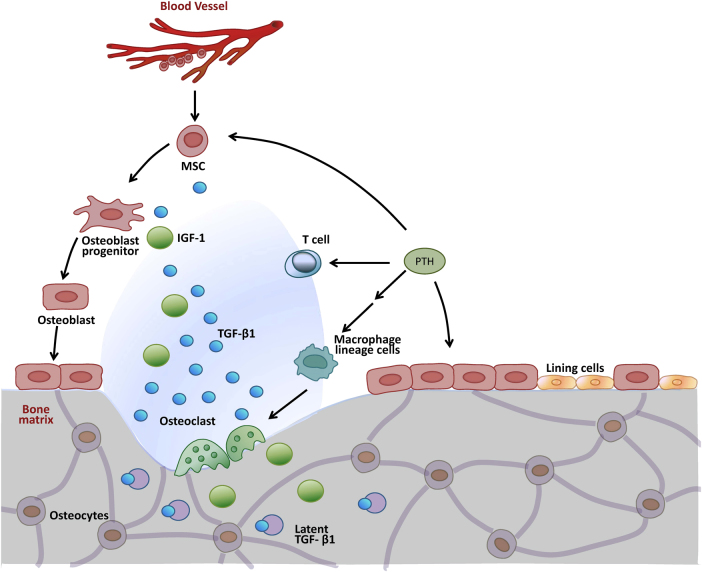
Activation of TGF-β recruits mesenchymal stem cells (MSCs) during bone remodeling. TGF-β1 is released from the bone matrix and activated during osteoclast-mediated bone resorption, creating a gradient. TGF-β1 induces migration of MSCs to the bone remodeling sites to couple bone resorption and formation. The bone-resorptive microenvironment also provides signals (e.g., IGF-1) that direct the lineage-specific differentiation of MSCs. In addition, PTH orchestrates signaling of local factors and thus regulates cellular activities, including those of MSCs, T cells, and other PTH-responsive cells in the bone marrow to coordinate bone remodeling

To overcome the challenge during evolution of vertebrate animals transitioning from ocean to land, cytokines and growth factors were likely selected to deposit in the bone matrix during bone formation. A large reservoir of factors in the bone matrix becomes available during osteoclastic bone resorption to induce recruitment and differentiation of MSCs and limit further osteoclastic resorption, thereby coupling bone resorption and formation. A critical step of coupled bone remodeling is to recruit MSCs to the bone resorption sites. Vertebrate TGF-βs 1–3, which appear to have no counterparts in nematodes (*Caenorhabditis elegans*) or insects (*Drosophila*),^327^ may have evolved to serve as the major coupling factor that coordinates the dynamic bone remodeling process of vertebrate mammals after they transitioned from ocean to land (Fig. 2). A more complex mechanical loading to the terrestrial vertebrates may promote the release of active TGF-β from the bone matrix^328^ for a coupled bone remodeling. In addition, osteoclastic bone resorption evolved by terrestrial vertebrates have an essential role in activating TGF-β during bone remodeling. Osteoclasts can form an integrin-dependent sealing zone between the bone and the cell,^106^ generating an acidic environment ideal for the activation of latent TGF-β richly deposited in the bone matrix.^37,107–109^ Osteoclasts can also activate latent TGF-β by secretion of proteases in the absence of a low-pH environment,^109^ leading to the proteolytic cleavage of LTBP1^329^ and enrichment of active TGF-β within the immediate environment of the bone resorption site. More importantly, our recent work has clearly demonstrated that TGF-β1, as one of the most abundant bone matrix cytokines,^330^ is activated during osteoclastic bone resorption and induces migration of MSCs to the bone remodeling sites via Smad-dependent signaling.^8^ TGF-β can also promote the migration of MSCs via JNK pathway and the induced expression of MCP-1, although these findings have only been reported in an artery injury model.^224^ In parallel, other growth factors released from bone matrix during resorption, such as IGF-1, promote osteoblast differentiation of MSCs.^222^ Studies of the effects of TGF-β1 on osteoclast activity show that high concentrations and prolonged exposure to active TGF-β1 inhibit migration of osteoclast precursors (macrophages/monocytes).^331^ Hence, the gradient of TGF-β1 generated at the resorptive site inhibits further recruitment of osteoclast precursors and avoids excessive bone resorption. This is particularly important for coupled bone remodeling since continuously elevated osteoclastic activity reduces the quality of bone and results in pathologic conditions.

Interestingly, osteoblast bone formation still exists in vertebrates that have defective osteoclasts function to release sufficient bone matrix-derived growth factors, indicating that osteoclasts per se may also be able to produce coupling factor(s).^332^ These potential coupling factors released by osteoclast include Sphingosine 1-phosphate, platelet-derived growth factor-bb (PDGF-bb), and the EphB4.ephrin-B2 bidirectional signaling complex. Sphingosine 1-phosphate is secreted by osteoclasts, and functions to recruit osteoblast and promote survival of mature osteoblast.^333^ Osteoclasts can also secret PDGF-bb, which not only induces migration and osteogenic differentiation of MSCs,^334^ but also controls osteoblast chemotaxis via PDGF-bb/PDGFR-beta signaling.^335^ More importantly, our recent findings have shown that PDGF-bb secreted by preosteoclasts is able to induces the formation of CD31(hi)Emcn(hi) vessel, a specific vessel subtype that functions to couple angiogenesis and osteogenesis during bone modeling and remodeling.^336^ EphB4 receptors are expressed on osteoblasts, whereas the ligand ephrin-B2 is expressed by osteoclasts. Signaling through EphB4 receptors induces osteoblastic differentiation, whereas signaling through ephrin-B2 inhibits osteoclastogenesis.^337^ The bidirectional signaling complex of EphB4.ephrin-B2 functions to activate osteoblastic bone formation and inhibit osteoclastic bone resorption simultaneously, and this is particularly critical at the transition point of bone remodeling. Notably, the direct cell contact between osteoblasts and osteoclasts within the BMU is not always possible, and osteoblastic bone formation could sustain long after osteoclasts vacate the resorption site. This fact suggests that both contact-dependent and contact-independent signals are involved in a coupled bone remodeling process.

### Bone remodeling dynamically changes the bone marrow microenvironment

The self-renewal, transient amplification, or differentiation of bone marrow MSCs are dynamically regulated by the bone marrow microenvironment.^338–340^ External signals produce transcription responses that allow cells to respond to cues from their environment at a certain magnitude and duration. In the reversal phase of bone remodeling, the bone-resorptive microenvironment provides signals that inhibit bone resorption and promote bone formation by recruiting MSCs and inducing osteoblastic differentiation. Multiple cytokines, growth factors, and minerals are released from the bone matrix or secreted by local cells during the bone remodeling. For example, IGF-1 released from the bone matrix can stimulate osteoblast differentiation of MSCs by activation of mammalian target of rapamycin (mTOR) through the PI3K/Akt pathway.^222^ Semaphorin 4D (SEMA4D), which is expressed on the cell surface of osteoclasts, binds to its receptor, Plexin-B1, on osteoblasts to inhibit the RhoA/Rho-associated protein kinase (ROCK) pathway.^341^ The ROCK pathway normally phosphorylates IRS-1, a key factor in the PI3K/Akt/mTOR pathway.^341^ Therefore, osteoclast expressing SEMA4D prohibits MSC differentiation via cell-to-cell contact, creating a boundary between bone resorption and formation. Thereby, the dynamic changes in the bone marrow microenvironment result in the coordination of the reversal phase during coupled bone remodeling.

In addition, the elasticity of the bone matrix has an important role, with a stiffer matrix directing differentiation of MSCs into osteoblasts.^330^ At fresh bone resorption sites, the bone mineral matrix without lining cells is exposed, providing a stiff elastic microenvironment in favor of osteoblastic differentiation. Blood vessels are in close proximity to bone remodeling, and a complex interrelationship between angiogenesis and osteogenesis has been reported.^342^ Basic fibroblast growth factor (FGF2) is a potent mitogenic factor that can promote angiogenesis by inducing endothelial cell proliferation, migration, and expression of necessary angiogenic factors including proteases, growth factors, and integrins.^343–346^ TGF-β has been shown to enhance production FGF2 in osteoblasts.^347–349^ In aortic endothelial cells, TGF-β stimulation of an ALK5/TβRII-Smad2 complex enhances expression of vascular endothelial growth factor (VEGF),^350^ which is a strong pro-angiogenic factor. Whether a similar mechanism of action exists in cells in the bone remodeling site remains to be investigated. HSCs are present in the same niche as MSCs.^351^ Although MSCs and osteoblast precursors can influence the fate of HSCs by producing several key maintenance factors including CXC chemokine ligand 12 (CXCL12), angiopoietin 1(ANGPT1), KIT ligand (KITL), and vascular cell adhesion molecule 1 (VCAM1),^323,351–353^ the opposite remains unknown.

### PTH serves as endocrine regulator of skeletal TFG-β to control bone homeostasis in terrestrial vertebrates

The parathyroid gland evolved in amphibians,^354^ and represents the transition of aquatic to terrestrial life. PTH is the major endocrine hormone produced by the parathyroid gland, which regulates calcium homeostasis. Interestingly, permanent emergence of osteoclasts and bone resorption is also observed as vertebrates transitioned to land, ^297–300^ and favors survival by lightening skeletal weight and a ready source of calcium. In addition to the critical role of PTH mobilizing calcium in times of need, PTH has also been shown to orchestrate signaling of local bone factors including TGF-β, Wnts, BMP, and IGF-1, integrating systemic control of bone remodeling.^185,355–362^ (Fig. 2)

PTH orchestrates the signaling of many local factors that determine the fate of MSCs. Endocytosis of growth factors and G protein-coupled receptors is known to integrate different signaling pathways.^363^ PTH can induce the recruitment of TβRII as an endocytic activator.^185^ TβRII directly phosphorylates the cytoplasmic domain of the PTH type 1 receptor (PTH1R) and facilitates PTH-induced endocytosis of a PTH1R-TβRII complex, resulting in downregulation of TGF-β effects, likely limiting further MSC recruitment. Concomitantly, PTH stimulates the commitment of MSCs to the osteoblast lineage by enhancing bone morphogenetic protein (BMP) and Wnt signaling. Low-density lipoprotein-related protein 6 (LRP6) serves as a co-receptor in the canonical Wnt pathway^364^ and interacts with BMP signaling.^365^ PTH has been shown to recruit LRP6 as a co-receptor, which recruits axin from the cytoplasm to stabilize β-catenin.^360^ PTH binding to PTH1R can also induce endocytosis of a PTH1R/LRP6 complex, resulting in enhanced BMP-pSmad1 downstream signaling, and ultimately promotes osteoblastic differentiation of MSCs.^362^ Deleting LRP6 in mature osteoblasts in mice results in a loss of anabolic response to PTH.^366^ PTH can expand Nestin-positive MSC populations,^323,352,367^ although the precise mechanism of action remains unclear. PTH-enhancement of MSC transient amplification, differentiation, and function is a part of the integration of the signaling networks of local factors for the spatial-temporal regulation of bone remodeling.

PTH can modify other cells in the bone marrow microenvironment. Intermittent PTH treatment increases the number of osteoblast by converting lining cells to active osteoblasts.^368^ PTH stimulates bone marrow CD8^+^ T cells to produce large amounts of Wnt10b, which activates Wnt signaling in MSCs and osteoblast precursors, thus increasing osteoblast proliferation and differentiation.^369^ PTH has also been shown to improve the bone marrow microenvironment by spatially relocating small blood vessels in proximity to sites of new bone formation, possibly via upregulation of VEGF-A and neuropilin-1 and -2.^370^ The consequent proximity of blood vessels allows more efficient delivery of nutrients to support new bone formation.

Taken together, TGF-β is a critical factor in the temporal and spatial coupling of bone remodeling. PTH, the hormone with evolutionary significance, may act as the major endocrine regulator of bone remodeling by orchestrating the signaling of many pathways and directing the fate of MSC.

## Aberrant TGF-β signaling leads to multiple pathologies

Temporal and spatial regulation of TGF-β activation is essential in maintenance of tissue homeostasis and regeneration of the damaged tissue by recruiting stem/progenitor cells to the right place at the right time, whereas sustained abnormal activation of TGF-β may lead to pathological conditions due to excessive recruitment of stem/progenitor cells and their subsequent differentiation. Here, we discuss multiple pathologies associated with abnormal TGF-β signaling. Typical skeletal disorders are taken as examples to demonstrate how abnormalities of TGF-β signaling lead to loss of site-directed recruitment of MSCs that causes uncoupled bone remodeling phenotypes. Other diseases with well-documented TGF-β involvement, such as tissue/organ fibrosis and bone metastases of cancer are also discussed in this section.

### TGF-β-induced osteoid islets in the subchondral bone initiate osteoarthritis

Osteoarthritis (OA) is a degenerative joint disease characterized by progressive articular cartilage degradation, subchondral bone sclerosis, reduced mobility, and debilitating joint pain. Although it is still under debate whether OA starts in cartilage or bone,^371^ subchondral bone sclerosis has now been recognized as a major contributor to the cartilage degeneration by generating unevenly distributed stress on the articular cartilage, leading to its gradual deterioration.^372,373^ Recent evidence has demonstrated that the onset of OA is associated with increased bone remodeling in the early stage,^374,375^ and subsequent slow turnover/densification of the subchondral plate and complete loss of cartilage.^376–378^ Notably, subchondral sclerosis without precedent stage of increased bone remodeling does not lead to progressive OA in experimental models.^376,379^ Therefore, both early stage increased bone loss, and the late-stage subchondral densification are important for the pathogenesis of cartilage degeneration in OA. Importantly, our recent findings have shown that this spatial and temporal separation of subchondral bone phenotype is likely initiated by the abnormal activation of TGF-β.^6^

Bone is constantly remodeled or modeled in response to changes in mechanical loading, particularly, when joint stability is decreased such as occurs during aging or with ligament injury or obesity. As osteocytes are the principal mechanosensors, aberrant mechanical loading may result in alterations in the release of signaling molecules such as OPG, RANKL, and sclerostin to increase osteoclast activity and turnover rate in OA subchondral bone. In post-surgery of anterior cruciate ligament transection (ACLT) OA mice, osteoclastic bone resorption in the subchondral bone was significantly increased as early as 7 days. The excessive release of active TGF-β1 to the bone marrow caused by osteoclastic bone resorption recruits Nestin^+^ MSCs to form marrow osteoid islets. Notably, osteoclastic bone resorption was temporally and spatially separated from TGF-β1-induced recruitment of MSCs in the marrow, resulting in aberrant bone formation. The uncoupled bone remodeling process in subchondral bone alters its micro-architecture and eventually compromises the functional integrity of “subchondral bone-articular cartilage complex”.^380^ The notion was further supported by the development of OA-like changes in the CED mouse model with aberrant activation of TGF-β1.^6^

Biomechanical factors have an essential role in the degenerative process of articular cartilage, which is maintained in a mechanically active environment. The subchondral bone volume and subchondral bone plate (SBP) thickness fluctuate substantially in ACLT rodent models. In human osteoarthritis joints, SBP is markedly thicker relative to those of healthy subjects.^376^ Computerized stimulation models of human knee OA suggest that expansion of 1–2% subchondral bone significantly changes the distribution of articular cartilage stress. In addition, the formation of osteoid islets and aberrant bone formation induced by TGF-β1 changes micro-architecture of subchondral bone.^6^ Although intermittent articular loading seems to be necessary for normal cartilage metabolism, abnormal loading patterns mediated by TGF-β1 may irregularly induce progressive cartilage degeneration.^381^ Cell death, water content and fibronectin content in the cartilage explants are increased in a load duration and magnitude-dependent manner.^382^ Chondrocytes in the superficial zone are more vulnerable to repetitive mechanical loading than those in the deeper layer of articular cartilage.^383,384^ Vigorous cyclic loading also leads to cartilage matrix damage such as breakage of collagen fiber and proteoglycan depletion possibly due to increased MMP-3.^385^ Therefore, the fluctuation of the mechanical property of subchondral bone inevitably affects its capacity to dissipate the mechanical stimuli from the joint surface and consequently leads to cartilage degeneration in OA. The loss of cartilage integrity will in turn increase the overload of the joint, leading to subchondral bone sclerosis as the joint attempts to adapt to the increased loads. Ultimately, this positive feedback loop causes progressive deterioration of articular cartilage in clinically evident OA.^376^

In addition to induction of aberrant bone formation in the subchondral bone of OA, excessive activation of TGF-β can affect multiple joint tissues. TGF-β signaling is crucial in normal cartilage development and the maintenance of articular chondrocyte homeostasis in synovial joints.^386,387^ However, recent findings by van der Kraan et al.^388^ have shown that ageing process may switch the TGF-β signaling in chondrocytes from the canonical anabolic ALK5-Smad2/3 pathway to the catabolic ALK1-Smad1/5/8 pathway, suggesting that excessive activation of TGF-β in aged individuals might actually exacerbate cartilage deterioration. Excessive TGF-β also induces synovial fibrosis and osteophyte formation,^389,390^ which are common features of osteoarthritis and also closely associated with its progression.

### Genetic mutations in TGF-β signaling components cause bone-associated disorders

TGF-β and its family members are multi-functional growth factors that have critical roles in development and maintenance of the skeleton. Various pathological skeletal phenotypes are consequent to mutations in genes encoding ligands, receptors, and signaling molecules of the TGF-β family. The TGF-β related diseases form an important subgroup of skeletal dysplasia,^391^ which covers both monogenic and polygenic diseases involving the skeletal system. The monogenic skeletal disease is caused by a single gene mutation and belongs to relatively simple and traceable Mendelian disease. In contrast, the polygenic skeletal disease is multifactorial (e.g., osteoporosis). Its phenotype is determined by combined and concerted effects of a group of genes (susceptibility genes) and the environment. Hence, its inheritance is complex and less predictable. These diseases give us clues to delineate the roles of TGF-β in the skeleton in vivo as well as physiological mechanisms controlling the skeletal system. Here we discuss representative monogenic and polygenic bone disorders associated with TGF-β mutations with a focus on their significance for understanding mechanisms regulating the skeletal system.

#### Monogenic bone disorders

**Fig. 3:**
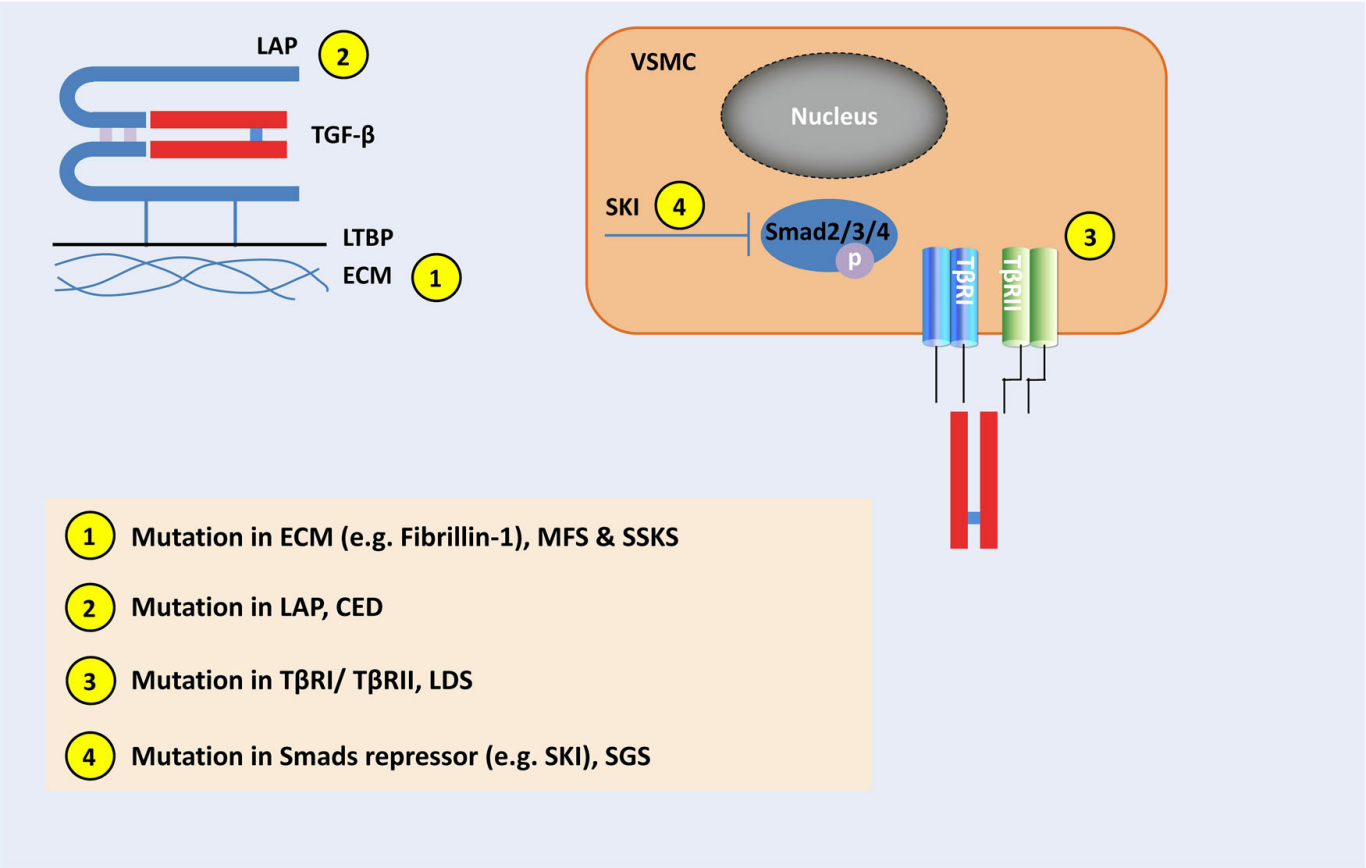
Common genetic disorders with aberrant TGF-β activity. ① Mutations in genes involved in the synthesis/assembly of extracellular matrix (ECM), e.g., Fibrillin-1 (FBN1), cause compromised matrix sequestration of the large latent complex of TGF-β and excessive TGF signaling, ultimately resulting in genetic disorders such as Marfan syndrome (MFS) and stiff skin syndrome (SSKS). ② Mutations in the region encoding latency-associated peptide (LAP) increase the release of active TGF-β, and cause Camurati–Engelmann disease (CED). ③ Mutations in genes encoding TGF-β type I and II receptors (TβRI/II) lead to compensatory synthesis of TGF-β ligand, and cause Loeys–Dietz syndrome (LDS). ④ Mutations in smads repressor, such as SKI, super-activate TGF-β signaling and causes Shprintzen–Goldberg syndrome (SGS) phenotypes. VSMC vascular smooth muscle cells

In general, most if not all monogenic TGF-β-related skeletal disorders share a common pathogenesis. A specific gene mutation leads to excessive and sustained production or activation of TGF-β ligand (Fig. 3), which distorts the normal bone remodeling process. The temporally and spatially regulated physiological TGF-β gradient may be disrupted and lead to excessive recruitment of MSCs or altered downstream targeted effects. Skeletal phenotypes with uncoupled bone remodeling appearing as sclerosis result in addition to other specific end-organ effects.

Camurati–Engelmann disease (CED) is characterized by a fusiform thickening of the diaphysis of the long bones and skull. CED is caused by mutations in the TGF-β1 gene, resulting in premature activation of TGF-β1.^392–395^ Approximately 11 different TGF-β1 mutations have been identified from CED-afflicted families.^396,397^ All mutations are in the region encoding LAP, which either destabilize disulfide bridging of LAP or affect secretion of the protein, leading to enhanced TGF-β1 signaling. Patients with CED show a typical uncoupled bone remodeling phenotype, characterized with decreased trabecular connectivity despite normal osteoblast and osteoclast numbers.^397,398^ The conditioned medium collected from cells expressing the CED mutant TGF-β1 shows significantly increased ratio of active/total TGF-β1, and it hyperactively induces the migration of MSCs.^8^ However, the targeted recruitment of MSCs to the bone remodeling site is likely disrupted due to loss of the TGF-β gradient.

In addition to CED, several other genetic disorders with skeletal manifestations including Marfan syndrome (MFS), Loeys–Dietz syndrome (LDS), Shprintzen–Goldberg syndrome (SGS), and neurofibromatosis type 1 (NF1) also involve aberrant TGF-β signaling. MFS is caused by mutations in the fibrillin-1 encoding gene (FBN1) and often results in aortic dilatation, myopia, bone overgrowth, and joint laxity.^399–402^ Fibrillin-1 is deposited in the ECM and interacts directly with latent TGF-β-binding proteins (LTBPs), keeping TGF-β sequestered and unable to exert its biological activity. In MFS, secondary to mutated structural fibrillin-1, excessive activation of TGF-β in the lungs, heart valves, and aorta causes the pathological features.^400,403^ LDS is caused by inactivating mutations in genes encoding TβRI and TβRII.^404^ Physical manifestations of LDS include arterial aneurysms, hypertelorism, bifid uvula/cleft palate and bone overgrowth resulting in arachnodactyly, joint laxity, and scoliosis. Although the exact mechanism is still unclear, TGF-β signaling is elevated in affected tissues of LDS despite the inactivating mutation.^404^ A recent study by Dietz and coworkers^405^ suggests that vessel smooth muscle cells (VSMCs) that carry the LDS mutation may compensate for their signaling deficiency by upregulating the expression of TGF-β ligands. Indeed, tissue from LDS patients or mice shows increased expression of TGF-β1 regardless of etiologies.^406,407^ The upregulated TGF-β1 production by specific cell types (e.g., VSMCs) could lead to paracrine overdrive of TGF-β signaling in neighboring cells despite the presence of mutant receptors. In addition, infiltrating CD45^+^ cells in the aortic root adventitia as the disease progresses, might also contribute to excessive TGF-β1 production.^405^ SGS is caused by mutations in the Sloan-Kettering Institute (SKI) proto-oncoprotein, ^408,409^ and results in similar physical features as MFS plus craniosynostosis. SKI negatively regulates Smad-dependent TGF-β signaling by impeding Smad2 and Smad3 activation, preventing nuclear translocation of the SMAD4 complex and inhibiting TGF-β target gene output by competing with p300/CBP for SMAD binding and recruiting transcriptional repressor proteins, such as mSin3A and HDACs.^410–412^ The neurocutaneous syndrome, neurofibromatosis type 1 (NF1), has skeletal features including kyphoscoliosis, osteoporosis, and tibial pseudoarthrosis. The *Nf1*^*flox/−*^*;Col2.3*^*Cre+*^ mouse model that closely recapitulates the skeletal abnormalities found in the human NF1 disease has hyperactive TGF-β1 signaling.^413^ The exact mechanisms underlying mutant neurofibromin-associated hyperactivation of TGF-β signaling remain unknown, particularly in relation to the osseous defects.

#### Polygenic bone disorders

Osteoporosis is the pathological decrease of bone tissue leading to an increased risk of fracture, mainly in spine (vertebral body), distal radius, and femoral neck. It has been estimated that osteoporosis results in more than 1.3 million osteoporotic fractures per year in the US, and more than 40% of postmenopausal women are reported to have sustained a fracture.^414,415^ Osteoporosis is classified as primary and secondary osteoporosis based on its etiology. Primary osteoporosis consists of postmenopausal and senile osteoporosis. Postmenopausal osteoporosis is the most common bone and joint disease in women after menopause. Secondary osteoporosis results from various diseases relating to bone metabolism, or prolonged use of medications such as glucocorticoids. The underlying mechanism in all cases of osteoporosis is an imbalance between bone resorption and bone formation. Three main mechanisms interplay and underlie the development of bone fragility, including an inadequate peak bone mass, excessive bone resorption, and inadequate bone formation during remodeling.^416^

Polymorphisms in several genes associated with bone mass or osteoporotic fracture have been identified by population-based and case-controlled studies. Candidate genes include TGF-β, vitamin D receptor (VDR), estrogen receptor (ER), and type I collagen.^417^ Specific TGF-β gene polymorphisms that are associated with BMD and/or osteoporotic fractures have been identified.^418,419^ A C/T polymorphism which causes a proline-leucine substitution at amino acid 10 in the TGF-β1 encoding region has been identified. The C allele is associated with increased BMD and a reduced osteoporotic fractures risk in postmenopausal Japanese women. In addition, the C allele is associated with circulating levels of TGF-β1, suggesting that the C allele may influence protein secretion or stability.^420^ However, the underlying molecular mechanisms are still unclear. Of note, subsequent large-scale association studies in Japanese^421^ and other ethnic populations^422,423^ have not been able to replicate the association.

### Sustained TGF-β activation results in organ fibrosis

Progressive fibrosis in tissues/organs, such as the liver, lung, kidney, heart, bone, and skin, is a major health burden and cause of patient suffering. It has been well-recognized that fibrogenesis is not a unique pathological process but the consequence of excessive tissue repair. A central event in tissue repair is the release of cytokines in response to injury. Accumulating data has evidenced that TGF-β can initiate and terminate tissue repair, and its sustained production/activation result in the development of tissue/organ fibrosis.

Excessive or sustained production/activation of TGF-β is a key molecular mediator of tissue fibrosis (Fig. 1). The pro-fibrogenic effect of TGF-β is mainly attributed to its capacity to attract fibroblasts and to stimulate their proliferation. Excessive activation of TGF-β also causes pulmonary and hepatic fibrosis by inducing EMT in alveolar epithelial cells and trans-differentiation of quiescent hepatic stellate cells into myofibroblasts, respectively.^424,425^ In addition, EMT may also contribute to TGF-β-induced cardiac fibrosis.^426^ Moreover, our recent data support the concept that TGF-β may promote the migration of either local resident MSCs or bone marrow MSCs to the injured tissues by local elevated levels of active TGF-β. The recruited MSCs can differentiate into myofibroblasts under sustained TGF-β stimulation, further contributing to the fibrosis/sclerosis of involved tissue/organs.^224,225^ Here, we elaborate on the specific roles had by TGF-β in the fibrosis of major organs.

#### Kidney

Kidney is particularly vulnerable to the consequences of fibrosis, largely due to its intricate anatomical architecture and filtrating function. Chronic kidney diseases (CKD) are characterized by the accumulation of ECM in the glomeruli (glomerulosclerosis) and/or the tubular interstitium (tubulointerstitial fibrosis). TGF-β has been identified as an important pathogenic factor in the course of CKD.

Accumulating evidence suggests a causal relationship between elevated level of TGF-β1 and the accumulation of ECM during glomerulosclerosis. In vitro incubation of normal glomeruli or mesangial cells with TGF-β1 led to accumulation of ECM characterized by increased production of extracellular-matrix proteins, suppressed protease activity, and increased integrin expression.^427–429^ Injection either a TGF-β1 neutralizing antiserum or a proteoglycan that binds TGF-β1 in nephritic rats prevented the increased production of matrix proteins by the glomeruli and blocked the accumulation of matrix.^430,431^ More importantly, overexpression of the TGF-β1 gene in rat kidneys led to rapid development of glomerulosclerosis.^432^ TGF-β can cause podocytopenia by inducing podocyte apoptosis and detachment from the glomerular basement membrane, thus initiating the development of glomerulosclerosis.^433,434^ TGF-β also induces mesangial expansion caused by mesangial cell hypertrophy, proliferation (and eventually apoptosis), and ECM accumulation.^435–437^ TGF-β also induces endothelial-to-mesenchymal transition (EndoMT) of glomerular endothelial cells through the Smad3 pathway,^438–440^ giving rise to glomerular myofibroblasts, a major source of ECM.

Tubulointerstitial fibrosis is characterized by excessive accumulation of ECM in the tubular interstitium, and is considered a central event in the progression of CKD, regardless of etiology. In addition, TGF-β mediates several key tubular pathological events, including fibroblast proliferation, EMT in tubular epithelial cells, fibroblast ECM production, and epithelial cell death, cumulatively leading to interstitial fibrosis.^441,442^

Myofibroblasts have been recognized as the dominant collagen-producing cells in organ fibrosis.^443,444^ A recent study elegantly performed by LeBleu et al.^445^ has delineated the origin of myofibroblasts in renal fibrosis. It has been shown that 50% of the myofibroblasts in renal fibrosis arise from local resident fibroblasts, 35% from bone marrow mesenchymal cells, 10% from the endothelial-to-mesenchymal transition, and 5% from the epithelial-to-mesenchymal transition program. Importantly, targeted knockout of Tgfbr2 in αSMA^+^ cells significantly reduced the myofibroblasts derived through differentiation from bone marrow cells, further indicating the role of TGF-β in the recruitment of bone marrow MSCs and the induction of their differentiation into myofibroblasts.

#### Liver

Liver fibrosis is the final consequence of many chronic liver injuries/diseases^446^ such as cirrhosis and hepatocellular carcinoma, which are leading causes of morbidity and mortality worldwide. Regardless of the inciting etiology, all the chronic liver diseases converge on a common pathophysiology, progressing from moderate to severe inflammation, next to fibrosis and finally to cirrhosis.

Multiple studies suggest that TGF-β is involved in many kinds of chronic liver disease (CLD). In liver-biopsy CLD specimens, the amount of TGF-β1 mRNA is positively correlated with that of type I collagen mRNA.^447^ TGF-β1 mRNA concentrations in the liver positively correlate with serum concentrations of peptide fragments of type III collagen and the histological activity of the liver disease. More importantly, TGF-β1 protein is specifically detected by immunohistochemical staining in areas of fibrosis.^448^ A similar pathophysiologic effect of increased levels of TGF-β occur in induced animal models for hepatic fibrosis.^449^ In a conditional transgenic mouse model with tetracycline-regulated expression of TGF-β1 in liver, fibrosis progressed to an intermediate state during the upregulation of TGF-β1 expression.^450^ Furthermore, the fibrogenic process can be attenuated simply by blockade of TGF-β signaling.^451^

TGF-β is believed to trigger a series of important cellular events related to fibrogenesis and repair in liver diseases.^452,453^ Most liver cells are sensitive to TGF-β, which initiates both the canonical Smad-mediated and the non-canonical Smad-independent downstream signals. In the early stages of liver fibrogenesis, excessive activation of TGF-β may impair liver regeneration^454^ and amplify hepatocyte apoptosis.^455^ Hepatic stellate cells are then activated and initiate fibrosis.^456^ During the development of fibrosis, TGF-β directly activates the quiescent hepatic stellate cells through the canonical Smad-dependent pathway, inducing its trans-differentiation into myofibroblasts, which deposit excessive ECM.^457^ TGF-β also has an essential role during the inflammatory process linked to liver fibrosis by mediating the terminal differentiation of regulatory T cells, negative regulators of inflammation. TGF-β induces cell death and EMT of hepatocytes, further contributing to the ECM deposition and fibrosis. Activation of liver sinusoidal endothelial cells and neoangiogenesis are also partially induced by TGF-β. TGF-β can induce a feed-forward loop through the production of ROS^458^ and eventually contributes to liver fibrosis.

#### Lung

The involvement of TGF-β in pulmonary fibrosis has been evidenced in both animal models and humans with pulmonary diseases. Rats induced with bleomycin for the development of fibrosis demonstrate significantly elevated levels of total lung TGF-β1 compared to the non-induced controls. Importantly, increases of TGF-β1 precede the synthesis of collagens, fibronectin, and proteoglycans.^459^ In people with idiopathic pulmonary fibrosis, TGF-β1 is increased in alveolar walls at the sites where ECM is accumulated.^460^ In addition, TGF-β mRNA level in bronchoalveolar cells obtained from patients with autoimmune diseases and lung fibrosis is 10 time higher than bronchoalveolar cells of normal subjects or patients with asthma.^461^ Alveolar macrophages are believed to produce the majority of TGF-β1 during the process of fibrogenesis.^462^ The increased TGF-β level in lung tissue can induce the trans-differentiation of resident cells to myofibroblasts, which secret the most ECM during fibrosis. Our recent data reveals that excessive TGF-β1 produced by allergen-challenged lung tissue can be released into circulation, and it mobilizes and recruits bone marrow MSCs to the perturbed airways. Those recruited MSCs may eventually differentiate into myofibroblasts under sustained TGF-β1 stimulation, further contributing to the lung fibrosis.^225^

#### Skin

Skin fibrosis is usually observed as a local manifestation of systemic connective tissue disorders such as systemic scleroderma,^463^ or a complication secondary to skin injury such as hypertrophic scars from burns.^464^ Regardless of etiology, increased amounts of TGF-β1 have a critical role in skin fibrosis. Of note, a self-limited acute injury will only elicit a transient activation of TGF-β for a desired action; whereas a repeated injury may override the normal termination signals of TGF-β activation, creating a sustained increase in TGF-β signaling, and blindly lead to the progressive deposition of ECM and skin fibrosis by either stimulating resident fibroblasts proliferation, or inducing trans-differentiation into myofibroblasts through EMT process.^465^

Sustained activation of TGF-β1 signaling caused by genetic mutations can result in familial skin fibrosis such as stiff skin syndrome (SSKS), which is characterized by hard, thick skin, limited joint mobility, and flexion contractures. Lipodystrophy and muscle weakness have also been reported occasionally.^466^ SSKS is caused by mutations specifically localized to the fourth transforming growth factor-β-binding protein-like domain (TB4) of fibrillin-1, which encodes the RGD motif, through which fibrillin-1 binds cell-surface integrins αvβ3, α5β1, and αvβ6.^466,467^ Recent findings from Dietz’s group have demonstrated that mouse lines harboring analogous amino acid substitutions in fibrillin-1 recapitulate aggressive skin fibrosis that is prevented by integrin-modulating therapies and reversed by antagonism of TGF-β.^468^ The proposed mechanism is that SSKS mutations promote increased deposition of abnormal microfibrillar aggregates that fail to make contact with neighboring cells but retain the ability to bind to the TGF-β LLC, resulting in an increased concentration of latent TGF-β in tissues of SSKS.^466,467^ The high dermal concentration of TGF-β in SSKS may then allow a sustained enhanced signaling state, possibly by mechanical traction-based activation of the excessive amounts of latent TGF-β in the stiffened dermis, resulting in a similar feed-forward mechanism as observed in fibrosis.^469^

TGF-β functioning as a key factor in fibrogenesis offers a promising target for the development of new therapeutic agents for the fibrotic conditions associated with excessive production/activation of TGF-β. Injection of TGF-β neutralizing antibody has been proven to be effective in the treatment of fibrosis in kidney,^430^ skin,^470^ lung,^471^ brain,^472^ joint,^473^ and arterial wall.^474^ Injection of TGF-β neutralizing antibody significantly reduced the synthesis of matrix proteins and the deposition of plasminogen activator inhibitor-type I in the glomeruli, and blocked the accumulation of mesangial matrix in nephritic rats. Treatment with TGF-β1 neutralizing antibody also substantially reduced the collagen content of dermal wounds and minimized the scar formation. Anti-TGF-β1 treatment also worked well at the site of brain injury by reducing fibrous scar tissue and inflammation. Anti-TGF-β1 could alleviate synovial inflammation and delay bone and synovial destruction in arthritic joints. In addition, injection of anti-TGF-β1 neutralizing antibody to the rats with carotid-artery injury suppressed the accumulation of matrix that underlies the development of intimal hyperplasia and restenosis. Similarly, our recent data obtained from mouse model of wire-induced injury of femoral artery have shown that intravenous administration of TβRI inhibitor (SB-505124) significantly diminished the formation of neointima in injured arteries compared to vehicle-treated control.^224^ These consistent therapeutic successes in animal models underscore the translational potential of TGF-β1 modulation in the treatment of organ fibrosis.

### Bone matrix-derived TGF-β promotes skeletal metastases

Cancer diagnosed at an early stage can usually be effectively treated with some combination of surgery, radiation, hormonal, and chemotherapies. However, treatment options for metastatic or recurrent cancer with acceptable outcomes are still limited. Bones are a common place for metastasis. Skeletal metastasis of cancer is a complex process, including cancer cells escaping the primary site, circulating to distant sites, evading the host immune response, and proliferating into the metastatic site.^475^ Tumor cells could generate adhesive molecules that mediate binding to marrow stromal cells and bone matrix. The interactions between tumor cells and bone promote the production of angiogenic and bone resorbing factors by tumor, and further enhance tumor growth in bone.^476^ Of note, the bone microenvironment houses abundant growth factors including TGF-β, IGF-1, and -2, FGFs, PDGFs and BMPs.^477^ During tumor-induced osteoclastic bone resorption, these factors are released and enriched in the bone microenvironment. These bone matrix-derived factors, particularly TGF-β, can act back upon the tumor to facilitate further tumor expansion and enhance cytokine production, and also upon osteoblasts to suppress bone formation, further promoting tumor growth and metastasis in bone.^478^

In cancer, TGF-β can be either a tumor suppressor or a promoter depending on the temporal stage of the disease.^479^ In the early stage of tumors initiation, TGF-β limits the growth of tumor cells through its antiproliferative and proapoptotic actions.^480^ Conversely, during tumor progression, TGF-β acts as a tumor-promoter by inducing proliferation, angiogenesis, and immunosuppression, and thus promotes invasion and metastasis of cancer.^481^ The loss of function of TGF-β signaling also contributes to certain tumor types. Mutations in TβRII are frequently detected in colon cancer, gastric tumors, and gliomas. These mutations may result in DNA repair defects and cancer predisposition, likely due to cellular escape from TGF-β-mediated growth surveillance.^482–485^ Loss-of-function mutations in TβRI have been observed in ovarian cancers, metastatic breast cancers, pancreatic carcinomas, and T-cell lymphomas.^486–489^ In addition, mutations in the TGF-β signaling components, such as Smad proteins mutations have been detected in several carcinomas. Smad2 mutations have been identified in human colorectal cancer and lung cancers.^490,491^ Smad4 mutations have been detected in pancreatic carcinomas and familial juvenile polyposis,^492,493^ and Smad2 and Smad4 double mutations have been detected in hepatocellular carcinoma.^494^ Moreover, Smad3 is frequently downregulated in cancer and inactivating mutations have also been reported.^495–497^ TGF-β can antagonize BMP-like responses by the formation of pSmad1/5–pSmad3 complex specifically binding to BRE and suppressing the transcription of downstream genes.^155^ Inactivation of Smad3 in cancer alleviates the antagonism of TGF-β on BMP responses, and enables TGF-β to induce BRE downstream ID genes,^155^ which are potent tumor promoters normally suppressed by canonical TGF-β signaling.^481^ The induction of ID genes thus deregulates tumor cell proliferation and confers invasiveness, angiogenesis, and metastasis.^498^

In advanced cancer, cells lose TGF-β suppressive effects resulting in compensatory over-production of TGF-β. Elevated level of TGF-β in serum is often observed in the later stages of cancer patient, and is associated with increased invasiveness and a poor clinical outcome of cancer.^499^ TGF-β signaling can act on multiple cells in the local microenvironment of bone, and consequently enhance tumor growth and invasiveness.^479,500^ Specifically, TGF-β could induce the expression, secretion and activation of MMPs that mediate the migration of endothelial cells, thus promoting tumor angiogenesis.^500,501^ TGF-β could also indirectly induce the expression of the pro-angiogenic factors such as VEGF and connective tissue growth factor (CTGF) in fibroblastic and epithelial cells,^502,503^ and thus further contributes to the angiogenesis and invasiveness of tumor.^504,505^ Inhibition of TGF-β signaling with TGF-β-neutralizing antibodies can suppress angiogenesis in human breast and prostate cancer,^506^ further validating the critical role of TGF-β as a pro-angiogenic factor during tumor. Furthermore, excessive production of tumor-derived TGF-β could suppress T-lymphocytes and natural killer cells, leading to cellular escape of cancer cells from cytotoxic T lymphocyte clearance.^507^ Target blockade of TGF-β signaling in T-cells results in eradication of tumors in mice challenged with live tumor cells,^508^ indicating the suppressive action of TGF-β signaling on the T-cell mediated antitumor immunity.

TGF-β is also involved in the EMT of cancer,^7,509^ which is essential to increase tumor cell mobility and invasiveness closely related to metastasis. TGF-β interacts with other oncogenic pathways to maintain the mesenchymal phenotype of tumor cells by downregulating E-cadherin and upregulating mesenchymal genes.^510^ Smads, Ras, Rho, ERK MAPK, p38 MAPK, and Wnt signaling pathways have been implicated in the TGF-β-induced EMT.^7^ TGF-β activates transcriptional factors such as Snail and Slug to regulate EMT.^511,512^ Particularly, SNAIL could repress E-cadherin and activate the transcription of mesenchymal genes, such as vimentin and αSMA. In addition, SNAIL could promote collagen I synthesis/deposition and upregulate pro-inflammatory interleukins such as IL-1, -6, and -8,^513,514^ and thus enhances the invasiveness of tumor.

On the basis of the aforementioned oncogenic activity of TGF-β, a feed-forward loop has been proposed to describe skeletal metastasis. Tumor cells in bone secrete osteolytic factors, such as PTHrP and IL-11, leading to osteolytic bone resorption. Active TGF-β is released from bone matrix by osteoclastic resorption and further induces tumor production of osteolytic and pro-metastatic factors including PTHrP and IL-11. Of note, PTHrP is a central mediator of TGF-β-induced osteolytic metastases. Increased expression of PTHrP has been observed in human breast cancer with bone metastases compared with primary breast cancers.^515^ PTHrP can stimulate RANKL and inhibit OPG expression in osteoblasts to favor osteoclastogenesis.^516^ TGF-β induces PTHrP secretion from MDA-MB-231 cells via Smad and p38 MAP kinase pathways.^517^ Stable inactivation of TβRII in the breast cancer cell line MDA-MB-231 inhibits TGF-β-induced PTHrP secretion and suppresses bone metastases in a mouse model.^518^ Direct neutralization of PTHrP with PTHrP-neutralizing antibodies inhibits development and progression of breast cancer bone metastases in mouse models.^519^ TGF-β released during bone resorption can also directly act on bone cells. Within a given range of concentrations, TGF-β stimulates osteoclastic bone resorption while inhibits osteoblastic differentiation. Transcriptional profiling of human breast cancer cells with an aggressive bone metastatic phenotype has identified the upregulation of several genes, such as IL-11, CTGF, CXCR4, and MMP1, which are associated with bone metastases. These genes act cooperatively to cause skeletal metastasis by promoting homing to bone, angiogenesis and invasion of tumor. Notably, these genes are all regulated by TGF-β via the canonical TGF-β/Smad pathway in metastatic cells.^503,520,521^ Either inhibition of TGF-β signaling with small-molecule inhibitors or inhibition of bone resorption with bisphosphonate is effective in decreasing TGF-β signaling activity in the bone metastases.^522^ This indicates that TGF-β released by osteoclastic bone resorption is the major source of TGF-β acting on tumor cells in bone. Inhibition of either the TGF-β pathway or osteoclastic bone resorption may represent a novel therapeutic for the treatment of skeletal metastasis.

Several preclinical studies have shown that the TGF-β signaling pathway is a potential target for the inhibition of bone metastases. Knockdown of Smad4 expression in breast cancer cells reduces growth of bone metastases,^523,524^ whereas overexpression of Smad7 reduces bone metastases of melanoma.^525^ Small-molecule inhibitors of the TβRI kinase have been used to reduce bone metastasis through blockage of TGF-β signaling. Systemic administration of small molecule [3-(pyridine-2yl)-4-(4-quinonyl)]-1H pyrazole] that is able to inhibit TβRI kinase activity has been shown to effectively reduce the number and size of lung metastases and the incidence of skeletal metastases in experimental mice model of bone and lung metastasis.^526^ Systemic administration of Ki26894 (a TβRI kinase inhibitor) decreased skeletal metastasis and prolonged survival of a nude mouse model with bone metastasis.^527^ Consistently, preventive treatment with TβRI kinase inhibitor LY2109761 led to reduction of the number of bone lesions and skeletal tumor burden in bone metastatic mice model. Although LY2109761 was less effective in the treatment of established bone metastases,^522^ SD-208, a more potent TβRI kinase inhibitor, was effective in the treatment of mice with established bone metastases.^520^ The use of TGF-β neutralizing antibodies is another possible modality for the treatment of bone metastases.^528^ Treatment with a neutralizing pan-TGF-β antibody (1D11, Genzyme) has been shown to decrease metastases to the lungs in a transplantable 4T1 mice model of metastatic breast cancer,^529^ and reduce skeletal tumor burden in mice while also increasing the bone volume.^530^

Combination of anti-TGF-β therapies with other therapeutics is promising for the treatment of patients with bone metastases. Bone is a hypoxic microenvironment, and hypoxia-inducible factor 1α (HIF-1α) has been implicated in enhancing tumor growth and metastasis.^520,531^ Hypoxia also stimulates the expression of CXCR4 and DUSP1,^532^ whose upregulation is associated with bone metastases.^503^ TGF-β stabilizes HIF-1α by inhibiting its degradation.^533^ TGF-β and hypoxia signaling pathways in breast cancer cells are additive to induce VEGF and CXCR4.^520,533^ Inhibition of both TGF-β and hypoxia signaling pathways decreases bone metastases more than inhibition of either alone, resulting in enhanced osteoblastic activity and suppressed osteoclastic bone resorption as well as reduced tumor growth.^520^ Other possible therapies include halofuginone, a natural product derivative that inhibits TGF-β signaling possibly via induction of Smad7. Halofuginone treatment can significantly suppress osteolysis and skeletal tumor burden in mice with established bone metastases.^534^

In conclusion, bone is the most common site of cancer metastases. Active TGF-β released from bone matrix is the major component of the bone microenvironment, functioning to drive a feed-forward cycle of tumor growth and osteolysis in bone. Modulation of TGF-β signaling in cancer cells has been proven effective to decrease bone metastases in either in vitro or animal models. Development of anti-TGF-β therapies administered alone or supplementing other therapies for bone metastasis of cancer is promising. As TGF-β can be either tumor suppressive or pro-metastatic, a long-term global blockade of this signaling pathway may result in off-target effects. Hence, a delicate modulation of TGF-β signaling during tumor onset and progression is necessary for the development of the most effective antineoplastic therapy with minimal toxicity but potent efficacy.

## Modulation of TGF-β signaling is promising for the treatment of disorders associated with TGF-β abnormalities

As the abnormalities in TGF-β signaling will cause a plethora of local or systemic disorders, the development of anti-TGF-β therapies is intriguing. Although many TGF-β targeting approaches have been developed and quite of few of these treatments have undergone clinical trials, concerns remain that long-term blockade of this pathway may have other off-target effects due to the dual functions of TGF-β in maintaining tissue homeostasis. It should always be kept in mind that a transient temporal and spatial activation of TGF-β is necessary for the maintenance of tissue homeostasis, whereas excessive production and sustained activation of TGF-β signaling will inevitably lead to TGF-β-mediated pathologies. Thereby, how to intricately tune the signaling to the optimal magnitude in the right place at the right time is becoming increasingly essential for TGF-β-targeted therapy. Here we discuss the recent updates in potential treatment for disorders with aberrant TGF-β signaling.

### TGF-β-modulation has shown significant clinical potential

Theoretically, every component of the TGF-β pathway can be a potential target for drug intervention. In reality, however, most of the treatments have not been validated in clinical trials yet. Currently, antibodies,^529,535,536^ antisense oligonucleotides (ASOs),^537–541^ ligand competitive peptides,^542–545^ and small-molecule inhibitors against particular component of the TGF-β pathway are being tested in clinic.

#### Monoclonal antibodies

Monoclonal antibodies against TGF-β can specifically neutralize excessive extracellular ligand. Monoclonal TGF-β1 antibody metelimumab (CAT-192),^546^ TGF-β2 antibody Lerdelimumab (CAT-152),^547,548^ as well as TGF-β1–3 pan-specific antibodies such as fresolimumab (GC-1008)^549,550^ have been well developed by Cambridge Antibody Technologies and Genzyme. Clinical trials on fresolimumab have been conducted for both neoplastic^551^ (ClinicalTrials.gov Identifier: NCT00923169) and non-neoplastic applications.^550^ (ClinicalTrials.gov Identifier: NCT01284322) Fresolimumab has been found to be well tolerated and safe at a single-dose infusion up to 4 mg/kg for the treatment of the fibrotic disorder focal segmental glomerulosclerosis,^550^ and at 15 mg/kg for the treatment of advanced malignant melanoma and renal cell carcinoma.^551^

Other antibodies that have been developed and tested in clinical trials include TβRII-blocking antibody and anti-integrin β6 antibody. Eli Lilly and Company developed a TβRII-blocking antibody, IMC-TR1, which is being evaluated in clinical trials for the treatment of breast and colon cancer (ClinicalTrials.gov identifier: NCT01646203). Another antibody against integrin β6 has also shown effectiveness in the treatment of fibrosis and cancer in animal models,^552^ and is currently in a Phase II clinical trial for the treatment of idiopathic pulmonary fibrosis (ClinicalTrials.gov identifier: NCT01371305).

#### Antisense oligonucleotides and antisense RNA

Antisense oligonucleotides have been developed to target mRNA translation thus downregulating ligand synthesis.^537,553^ Trabedersen, a synthetic 18-mer phosphorothioate-modified ASO, binds specifically to the human TGF-β encoding gene (TGFB2), and this drug has proven to be effective in the treatment of glioma. Preclinical and clinical studies.^537,538,540^ indicate that neutralization of TGF-β2-mediated immunosuppression can activate tumor-infiltrating natural killer cells, which suppress tumor proliferation. Trabedersen can bypass the blood–brain–barrier and achieve a homogeneous distribution throughout the tumor, resulting in shrinkage of the targeted tumor as well as tumors elsewhere in the brain. Phase I/II studies of trabedersen for the treatment of anaplastic astrocytoma (grade III glioma) and glioblastoma (grade IV glioma) showed survival benefit compared with conventional chemotherapy.^554^ More importantly, patients with glioblastoma on trabedersen treatment experienced less adverse events and showed significantly improved cognitive function 2–3 years after therapy compared to standard chemotherapy.^541^

One of the challenges of ASO is target-delivery to avoid off-target toxicity. In the case of glioblastoma, ASO was delivered directly into the tumor using an intrathecal catheter.^541^ Intravenous delivery of ASO has also been developed for pancreatic cancer in mouse models,^540^ as well as in humans.^555^ An anti-TGF-β2 antisense strategy has been used to augment tumor vaccines. Belagenpumatucel-L is such a tumor vaccine, in which a ~900-nucleotide TGF-β2 antisense construct is transfected into allogeneic non-small cell lung cancer (NSCLC) cells to enhance its tumor-suppressive efficacy. This tumor vaccine shows enhanced activity compared to conventional vaccines.^556,557^ A recent phase III study showed that Belagenpumatucel-L failed to improve the overall survival of the enrolled NSCLC patients compared to placebo, but an improved survival in patients who were randomized within 12 weeks of completion of chemotherapy and in those who had received prior radiation has been suggested. Further studies are still needed to validate the effectiveness of belagenpumatucel-L in the treatment of NSCLC.^558^

#### Ligand traps and peptides

Genzyme has developed a ligand trap by fusing Fcγ to the extracellular domain of TβRII. Although this construct can inhibit mammary tumor cell viability, migration, and metastases in the animal model,^559^ it has not been tested in clinical trials. A different ligand trap approach, using peptide mimetics of TβRIII (also known as betaglycan),^542–545^ has been investigated in a Phase IIa clinical trial for the treatment of scleroderma and skin fibrosis. Data from that trial show safety and efficacy of this ligand trap when topically applied to skin.

#### Small-molecule inhibitors

Small-molecule inhibitors (SMIs) are designed to specifically target the type I receptor of TGF-β to block the canonical Smad2/3 pathway while keeping other non-canonical responses such as TAK1 activation relatively intact. SMIs in general are ATP mimetics, which completely bind the hydrophobic ATP binding pocket of the receptor kinase.^560,561^ SMIs have proven to be effective in the control of cancer metastasis in preclinical studies.^562,563^ The advantages of SMIs include economical production, relative stability, and easy oral administration. A possible disadvantage of SMIs is the cross-inhibition of other kinases, resulting in off-target effects. The short half-life of SMIs favors a predictable inhibition of TGF-β signaling in a desired time frame, and provides the possibility of rapid drug withdrawal should adversary effects arise.

#### Pre-existing drugs that inhibit TGF-β signaling

Although the precise molecular mechanisms are still unclear, Losartan and Candesartan that are originally developed as angiotensin type II receptor inhibitors for the treatment of hypertension, are able to reduce aneurysm growth in both MFS and LDS likely by downregulating TGF-β signaling.^405,564^ In addition, angiotensin type II receptor inhibitors have also shown protective effect on patients afflicted with TGF-β over-activation-induced brain, lung, and muscle injuries.^565–567^ Pirfenidone, an approved drug for the treatment of idiopathic pulmonary fibrosis in Europe and Mexico, has shown inhibitory effect on TGF-β activity via unknown targets.^568^ Pirfenidone is currently in a Phase III trial in the United States.^569,570^

#### Other potential approaches

Other potential approaches to suppress TGF-β signaling include gene transfer of inhibitory Smad7, which has been tested in animal models for vascular remodeling, diabetic kidney disease, and colonic and hepatic fibrosis.^571,572^ The major concern that limits the clinical translation of this approach is the barriers that face all gene therapies.^573^ TGF-β activation may also be blocked by small peptides. An LSKL (Leu-Ser-Lys-Leu) peptide, which specifically binds to a conserved sequence in the LAP region of the TGF-β latent complex has shown effectiveness in suppressing TGF-β signaling in vitro.^71^ However, its TGF-β blockade efficacy has not been demonstrated in vivo yet.

#### TGF-β-modulation represents a promising treatment for osteoarthritis

Excessive activation of TGF-β in subchondral bone has been reported at the onset of OA in animal models.^6^ Active TGF-β1 concentrations are also high in the subchondral bone of people affected by osteoarthritis. All these data suggest that modulation of TGF-β signaling may provide a promising disease-modifying approach for OA. In the ACLT rodent models, systemic inhibition of TGF-β signaling with intraperiotoneal administration of TβRI inhibitor or specific blockade of active TGF-β ligand in the tibial subchondral bone with TGF-β neutralizing antibody at the time of injury was sufficient to attenuate the degeneration of articular cartilage.^6^ Furthermore, TGF-β signaling activated in the subchondral bone through osteoclast bone resorption in these animal OA models suggests that inhibition of osteoclast bone resorption may also delay progression of OA. Several clinical trials and preclinical studies support this hypothesis. In addition to many animal studies that reveal the positive effect of bisphosphonates for delaying OA progression,^574–578^ in the most recent prospective 2-year trial, alendronate treatment successfully improved the Western Ontario and McMaster Universities osteoarthritis (WOMAC) pain scores and decreased biochemical markers in hip OA patients.^579^ In a cross-sectional study, elderly women who were being treated with alendronate were found to have significantly decreased prevalence of knee OA, as assessed by WOMAC pain scales and subchondral bone lesions by MR imaging compared to elderly women not taking alendronate.^580^ Randomized controlled trials are needed to assess the efficacy of bisphosphonates as a potential OA treatment. The detailed mechanisms also still need to be further elucidated, anticipating that at least part of the effect is through inhibition of osteoclast activity and subsequent reduction in TGF-β levels. As many musculoskeletal disorders also involve excessive TGF-β signaling, attenuating the TGF-β signaling pathway could also benefit the management of these diseases.

High levels of active TGF-β also alter the microenvironment of subchondral bone, leading to aggregation of osteoprogenitors and increased angiogenesis in bone marrow. PTH is FDA-approved as an anabolic therapy for osteoporosis. Daily injection of PTH increases bone formation with normal micro-architecture.^185,355–362^ PTH can improve the bone marrow microenvironment by orchestrating the signaling of local factors for bone remodeling, reducing reactive oxygen species, and stimulating Wnt signaling in bone of senescent mice.^185,313,355–362,581,582^ In addition, PTH has demonstrated potent chondroprotective and chondroregenerative effects by inducing cartilage matrix synthesis and suppressing chondrocyte hypertrophy and matrix metalloproteinase 13 expression in different OA animal models.^583,584^ The dual beneficiary effects of PTH on both cartilage and subchondral bone make PTH another promising candidate for the treatment of OA. A recent study using a rabbit model of osteoarthritis preceded by osteoporosis (OPOA) has demonstrated that PTH treatment on the early stage of OA is able to improve microstructure and quality of subchondral bone, and thus attenuates subsequent cartilage damage.^585^ These data support the relevance of the role of subchondral bone osteopenia in the pathogenesis of OA, and indicate the potential application of anabolic agents (such as PTH) to the treatment in early stages of OA associated with osteoporosis. The osteogenic microenvironment created by PTH has also been shown to expand HSPC niches,^323,352^ and could also potentially be translated into therapies for hematologic diseases such as cytopenias, myelodysplastic syndromes, or myeloproliferative disorders.^586^

In conclusion, articular cartilage and subchondral bone constantly interact as a functional unit. TGF-β has a critical role in maintaining both bone and articular cartilage homeostasis. Joint instability causes increased subchondral bone remodeling, which releases excessive active TGF-β1 through osteoclastic bone resorption. The pathologically high level of TGF-β1 in the subchondral bone leads to aberrant bone remodeling and formation of marrow osteoid islets. The abnormal subchondral bone microstructure in turn likely alters the stress distribution on the articular cartilage, eventually resulting in cartilage degeneration. The concept of the holism is essential for exploring therapeutic strategies for OA. Improving mechanical properties of subchondral bone and its physiological function is at least equally important as directly targeting articular cartilage. Therapies that can normalize TGF-β signaling, either directly by neutralizing TGF-β over-activation or indirectly by PTH-mediated modulation of the bone marrow microenvironment, may serve as a potential approach to the management of joint disorders.

## Conclusion

TGF-β as a dual functional growth factor has been attracting major research efforts ever since its discovery three decades ago. Unlike most of the growth factors that are ready to function upon secretion, TGF-β is unique in that it is secreted as part of a latent complex that is stored in the ECM for activation and action at a later time point. Thereby, the magnitude and duration of TGF-β signaling are mainly dependent on, and meticulously controlled by the temporal and spatial activation of its ligand. If activated properly, TGF-β signaling has an essential role in normal physiology ranging from embryonic development to adult tissue homeostasis, whereas sustained activation or functional deletion via genetic mutations or environmental stimuli will exacerbate its adversary effects, and contribute to the pathophysiology of major diseases such as musculoskeletal disorders, cancer progression and organs fibrosis. Therefore, TGF-β has been described as “an excellent servant but a bad master”,^587^ in reference to its paradoxical characteristics.

Precise temporal and spatial activation of TGF-β signaling is necessary to counter tissue perturbations, either by suppressing excessive adversary cellular responses such as inflammation, or by recruiting adult stem cells to participate in tissues repair/remodeling process. The bone remodeling process clearly underscores the importance of proper activation of TGF-β in the maintenance of tissue homeostasis. The physiological gradient of active TGF-β formed by normal bone remodeling functions as a coupling factor that directs the MSCs/osteoprogenitors to the bone resorbing sites for a coupled bone formation, whereas sustained activation of TGF-β by either genetic mutations or pathologically elevated osteoclastic activity recruits excessive MSCs/osteoprogenitors for an aberrant bone remodeling. The same theory can be applied to other pathologies with TGF-β involvement, where the normal functions of this growth factor are switched to an off-target activity that disrupts tissue homeostasis.

Therefore, a comprehensive understanding of the mechanisms that underlie the physiological or pathological effects of TGF-β, and full interpretation of how cells integrate these signals into coherent responses in a context-dependent way, can lead to promising therapeutics for TGF-β-involved pathologies. Novel strategies that intricately tune TGF-β signaling to properly respond to specific contexts are being developed with anticipated clinical implications.
